# The TriTryp Phosphatome: analysis of the protein phosphatase catalytic domains

**DOI:** 10.1186/1471-2164-8-434

**Published:** 2007-11-26

**Authors:** Rachel Brenchley, Humera Tariq, Helen McElhinney, Balázs Szöőr, Julie Huxley-Jones, Robert Stevens, Keith Matthews, Lydia Tabernero

**Affiliations:** 1 Faculty of Life Sciences, Michael Smith, University of Manchester, M13 9PT, UK; 2 Computer Science, University of Manchester, M13 9PT, UK; 3 Institute of Immunology and Infection Research, University of Edinburgh, EH9 3JT, UK; 4 GlaxoSmithKline Pharmaceuticals, Essex, CM19 5AW, UK

## Abstract

**Background:**

The genomes of the three parasitic protozoa *Trypanosoma cruzi*, *Trypanosoma brucei *and *Leishmania major *are the main subject of this study. These parasites are responsible for devastating human diseases known as Chagas disease, African sleeping sickness and cutaneous Leishmaniasis, respectively, that affect millions of people in the developing world. The prevalence of these neglected diseases results from a combination of poverty, inadequate prevention and difficult treatment. Protein phosphorylation is an important mechanism of controlling the development of these kinetoplastids. With the aim to further our knowledge of the biology of these organisms we present a characterisation of the phosphatase complement (phosphatome) of the three parasites.

**Results:**

An ontology-based scan of the three genomes was used to identify 86 phosphatase catalytic domains in *T. cruzi*, 78 in *T. brucei*, and 88 in *L. major*. We found interesting differences with other eukaryotic genomes, such as the low proportion of tyrosine phosphatases and the expansion of the serine/threonine phosphatase family. Additionally, a large number of atypical protein phosphatases were identified in these species, representing more than one third of the total phosphatase complement. Most of the atypical phosphatases belong to the dual-specificity phosphatase (DSP) family and show considerable divergence from classic DSPs in both the domain organisation and sequence features.

**Conclusion:**

The analysis of the phosphatome of the three kinetoplastids indicates that they possess orthologues to many of the phosphatases reported in other eukaryotes, including humans. However, novel domain architectures and unusual combinations of accessory domains, suggest distinct functional roles for several of the kinetoplastid phosphatases, which await further experimental exploration. These distinct traits may be exploited in the selection of suitable new targets for drug development to prevent transmission and spread of the diseases, taking advantage of the already extensive knowledge on protein phosphatase inhibitors.

## Background

The parasitic protozoa *Trypanosoma cruzi*, *Trypanosoma brucei *and *Leishmania major *are the causative agents of human diseases known as Chagas disease, African sleeping sickness and cutaneous Leishmaniasis, respectively, that affect millions of people in developing countries (Central and South America, sub-Saharan Africa, Asia and parts of Europe) [1]. A major factor in the prevalence of these diseases is poverty and lack of medical resources in areas already afflicted by poor social support and threatened economies. Currently, there are no vaccines to prevent the spread of these diseases and many of the drugs available for treatment are highly toxic and require hospitalisation. In the past several years, new efforts have polarised the attention of laboratories worldwide to tackle the control of these neglected diseases. The genome sequences for these pathogenic kinetoplastids, and recently for two more *Leishmania *species, have now been reported [2-5] representing a major step forward to expand our understanding of their biology. This information provides a great opportunity to analyse particular gene families and to compare them with those of the host, with the aim to identify new targets for pharmaceutical intervention.

The sequenced kinetoplastid parasites have very different life cycles. Each parasite uses a different insect as their transmitting vector and prefers different conditions in which to survive and reproduce after infecting a human host. *L. major *is an intracellular parasite, seeking to invade macrophages and *T. cruzi*, also intracellular, invades and replicates in many cell-types, including macrophages and fibroblasts. *T. brucei *is an exclusively extracellular parasite that resides in the bloodstream of the mammalian host. As the parasitic life cycle takes these organisms through widely differing environments, frequent and substantial adaptive changes are required in many cell processes, resulting in changes in gene expression, protein levels and protein modifications [6-8]. A well-documented posttranslational modification is protein phosphorylation, which exhibits marked changes during development of these parasites [9,10].

Reversible protein phosphorylation is one of the most important biological mechanisms for the regulation of adaptive responses to intra- and extra-cellular signals in both eukaryotes and prokaryotes. Many cellular signalling pathways are modulated through the antagonistic activities of highly specific protein kinases and protein phosphatases that control a number of processes including metabolic pathways, cell-cell communication, cell growth and proliferation and gene transcription. Because of their essential roles, mutated forms of these proteins are often involved in disease states in mammals [11-14] or result in a severe reduction in virulence and infection of pathogenic bacteria [15-17]. The specific roles of protein phosphatases in unicellular protists, in particular protozoan parasites like Trypanosomes and Leishmania are less understood.

Recent work has identified several protein phosphatases and their roles in regulation of parasite development. *Tb*PTP1 phosphatase is a master switch in controlling differentiation in *T. brucei *[18]. In particular, this phosphatase inhibits the transition from the G0-arrested stumpy form in the blood stream to the procyclic form in the tsetse fly. *Tb*PTP1 inactivation by genetic or chemical means releases such inhibition and allows the cells to progress through differentiation to the procyclic form. Another protein tyrosine phosphatase, not orthologous to *Tb*PTP1, has been identified in *L. major *(*Lm*PTP1) that allows *L. major *amastigotes to survive in mice [19]. This may be an important factor in virulence, enabling the invading pathogen to survive in a host.

Other phosphatases characterised include PP1 from *T. cruzi *[20], PP1 and PP2A in *T. brucei *[21], PP5 in *T. brucei *[22] and protein phosphatases with EF-Hands (PPEF) in *T. cruzi*, *T. brucei *and *L. major *[23], each with various roles in the biology and development of kinetoplastids. All this work highlights the importance of protein phosphatases in the regulation of essential developmental aspects in the life cycle of pathogenic kinetoplastids.

Protein phosphatases are traditionally classified according to their substrate preferences, including serine/threonine phosphatases (STP), tyrosine-specific phosphatases (PTP), dual-specificity phosphatases (DSP), that dephosphorylate phospho-serine, phospho-threonine and phospho-tyrosine substrates, lipid phosphatases (PTEN type and Myotubularins), and the low molecular weight PTP (LMW-PTP). The presence of specific conserved motifs in the catalytic domain as well as additional regulatory or targeting domains allow these types to be recognised and classified into different subfamilies [24-27]. We have applied this knowledge on domain architecture and conserved catalytic motifs as the basis for the phosphatase domain classification.

A recent study of the TriTryp kinome [28] provides an overview of the kinase complement in the three parasites, and highlights important traits in the distribution of the different families of kinases. For a full understanding of phosphorylation-mediated events we also require knowledge of their counterpart protein phosphatases. We describe in this work the protein phosphatase complements of the TriTryp genomes. We employed an ontology-based classification tool that we have successfully used in the past to classify phosphatases in the Human and *Aspergillus fumigatus *genomes [27] to compile the phosphatomes of *T. cruzi*, *T. brucei *and *L. major*. We found that these organisms have an unusual composition of phosphatases with the PTP family being greatly reduced while the STP family has expanded by comparison with human phosphatases. We have also identified novel domain architectures in several phosphatases with potentially new functions and a number of unique and atypical phosphatases. With less than 30 phosphatases characterised in kinetoplastids out of more than 250, the information contained in the TriTryp phosphatome should stimulate further experimentation that would lead to a much more complete understanding on the biology of these important parasites. The significant divergence from human phosphatases indicates that these enzymes may be suitable targets for the development of specific inhibitors with therapeutic applications.

## Results and discussion

### The TriTryp phosphatome

**Figure 1:**
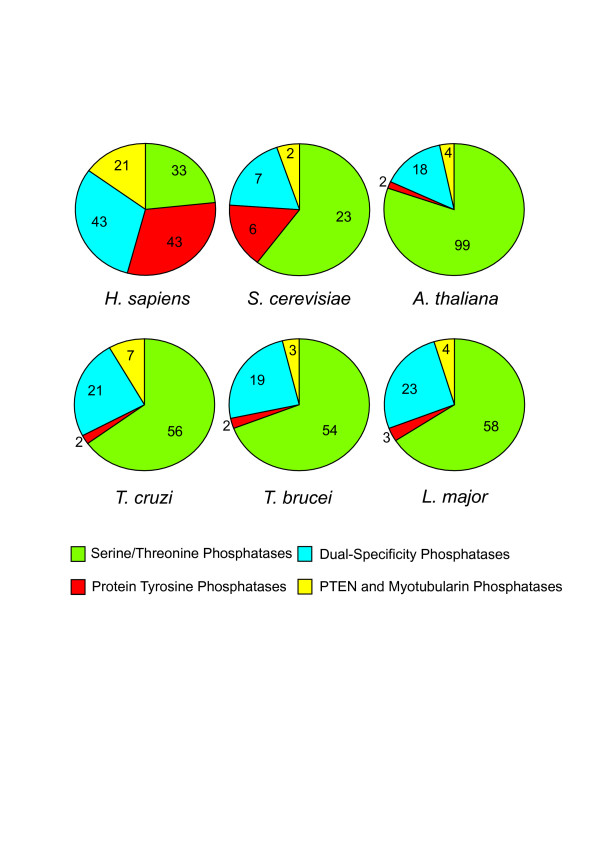
**Comparison of the protein phosphatase complement in different genomes**. Pie charts show the distribution of phosphatase catalytic domain genes in the different families: S/T Phosphatases, Protein tyrosine phosphatases, Dual-specificity phosphatases and PTEN/MTM lipid phosphatases. Phosphatase complements are shown for *T. cruzi*, *T. brucei*, *L. major*, in comparison with those for the Human [24, 26, 29, 128], *S. cerevisiae *[129, 130] and *A. thaliana *[76, 131] genomes. ACR2/cdc25-like are included in the DSP group.

Following the ontology classification, a total of 252 protein phosphatases were identified: 86 in *T. cruzi*, 78 in *T. brucei *and 88 in *L. major *(full list in Additional file 1). Overall there is a great deal of similarity across the three genomes regarding the number of phosphatases of each type (Figure 1, Table 1). The three kinetoplastids have representatives from all the major families of phosphatases: STPs, PTPs, DSPs and the lipid phosphatases PTEN and Myotubularins. Missing genes include the low molecular weight PTP (LMW-PTP) and the eyes absent (EYA) phosphatase. Consistent with the evolutionary divergence of kinetoplastid parasites, a high proportion of phosphatases were identified that have no clear orthologues in other genomes reported to date. We found that atypical and kinetoplastid-specific phosphatases amount to 36% for *T. cruzi*, 39% for *T. brucei *and a 41% for *L. major *of the total of phosphatases, most of them belonging to the STP and DSP subfamilies (Table 1).

**Table 1:** Total numbers of protein phosphatases from each subfamily in *T. cruzi*, *T. brucei *and *L. major*.

	*T. cruzi*	*T. brucei*	*L. major*
Protein Phosphatases (Total)	86	78	88
Protein Tyrosine Phosphatases (Total)	2	2	3
Classical PTP (Total)	2	2	3
**Eukaryotic-like**	**2**	**1**	**2**
**Kinetoplastid PTP (kPTP)**	**0**	**1**	**1**
Low Molecular Weight PTP	0	0	0
Dual-specificity phosphatases (Total)	20	19	22
**Eukaryotic-like**			
**Cdc14**	**1**	**1**	**1**
**PRL**	**3**	**1**	**2**
**Atypical-DSPs**			
**LRR-DSP**	**1**	**1**	**1**
**Kinatases**	**1**	**1**	**1**
**ANK-DSP**	**1**	**0**	**0**
**STYX**	**1**	**1**	**1**
**Other MKP-like**	**1**	**1**	**1**
**Lipid-like**	**2**	**2**	**4**
**Kinetoplastid-DSP (kDSP)**	**9**	**11**	**11**
PTEN (Total)	5	1	2
**Eukaryotic-like**	**4**	**0**	**1**
**Kinetoplastid PTEN (kPTEN)**	**1**	**1**	**1**
Myotubularin (Total)	2	2	2
**Eukaryotic-like**	**2**	**2**	**2**
Arsenate Reductases			
ArsC	1	1	1
ACR2 (cdc25-like)	1	0	1
Serine/Threonine phosphatases (Total)	56	54	58
PPP	29	27	30
**PP1**	7	8	8
**PP2A**	2	2	2
**PP2B**	2	2	2
**PP4**	1	1	1
**PP5**	1	1	1
**PP6**	1	0	1
**PP7(PPEF)**	2	2	1
**Kinetoplastid PPP (kPPP) incl.**	13	11	14
**Alphs**			
PP2C	14	13	15
**Eukaryotic-like**			
FCP	13	14	13
**Eukaryotic-like**			

The proportion of each subfamily of phosphatases is also significantly different from the human, *S. cerevisiae *and plant phosphatome (Figure 1). The most striking feature is the small proportion of PTPs present in the TriTryp phosphatome. This resembles only that of the plant *A. thaliana*. Whereas in humans and *S. cerevisiae*, PTPs comprise 31% and 16%, respectively of their phosphatase complement, *A. thaliana *and the kinetoplastids have a very small proportion: 2% for *A. thaliana *(2 proteins), 2.3% for *T. cruzi *(2 proteins), 2.6% for *T. brucei *(2 proteins) and 3.4% for *L. major *(3 proteins). When the proportion of STPs is analysed, there are also significant differences to the other species. *T. cruzi *has 65%, *T. brucei*, 69%, *L. major*, 66%, *S. cerevisiae*, 61% and *A. thaliana *81%, whereas the human genome only has 24%. The DSP family proportion in the kinetoplastids is similar to humans and *S. cerevisiae*: *T. cruzi *has 23% DSPs, *T. brucei*, 24% and *L. major*, 25% compared with 31% for the human DSPs and 18% in *S. cerevisiae*. *T. cruzi *has a higher proportion of PTEN and myotubularin phosphatases compared to the other two parasites, with 3 more PTEN sequences than *L. major *and 4 more than *T. brucei*. The low proportion of PTPs, together with the large proportion of STPs and of atypical and kinetoplastid-specific phosphatases, suggests that phosphorylation-mediated mechanisms in these parasites may have a different emphasis than in vertebrates. It is interesting that the extremely low proportion of PTPs seems to be compensated by an expansion of the STP family. This may be related to the absence of tyrosine-specific kinases encoded in the kinetoplastid genomes [28] whose activity may be replaced by dual-specificity protein kinases. Overall it seems that there is still much to uncover about the functions of phosphatases in organisms distantly related to the mammalian model, traditionally accepted as the standard.

### Protein tyrosine phosphatases

**Figure 2:**
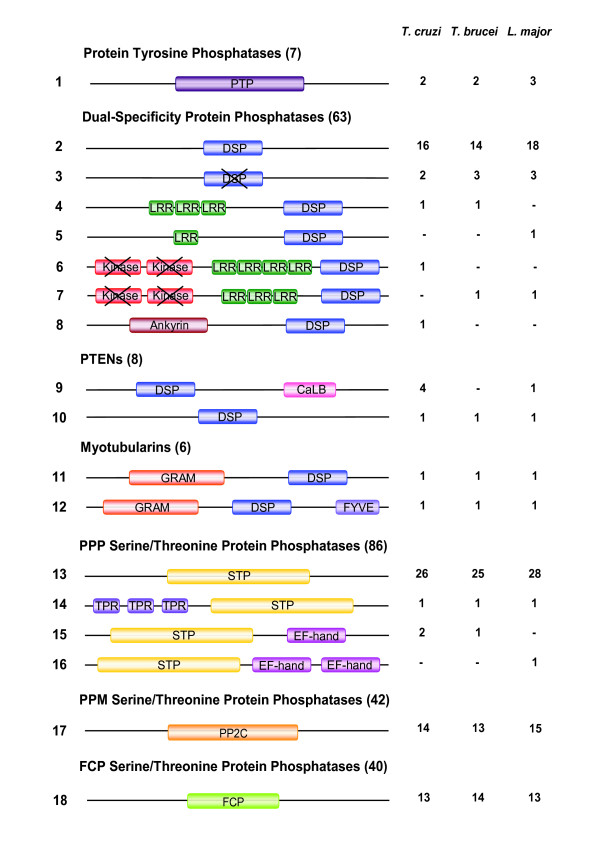
**Domain organisation of the *T. cruzi*, *T. brucei *and *L. major *phosphatases**. Domains are colour-coded according to the type of domain and grouped within phosphatase subfamilies. PTP, protein tyrosine phosphatase; DSP, dual-specificity phosphatase (crossed means pseudophosphatase); kinase, protein kinase domain (crossed means pseudokinase); TPR, Tetratricopeptide repeat; LRR, Leucine rich repeat; CaLB, Calcium lipid binding; GRAM, glucosyltransferases, Rab-like GTPases activators and myotubularins domain (plasma membrane protein-binding domain); FYVE, Fab1p/YOTB, Vac1p/EEA1 (PI3P binding domain); EF-hand, calcium binding domain; S/T phosphatase, serine/threonine phosphatase catalytic domain; FCP, CTD protein phosphatase. Note that many InterPro domains are variations representing the same biological function and sometimes they overlap. Only one domain is represented for these regions in this figure. Numbers of each domain type are listed for the kinetoplastids and '-' shows where one of the parasites lacks a particular architecture. ACR2/cdc25-like are included in the DSP group.

**Figure 3:**
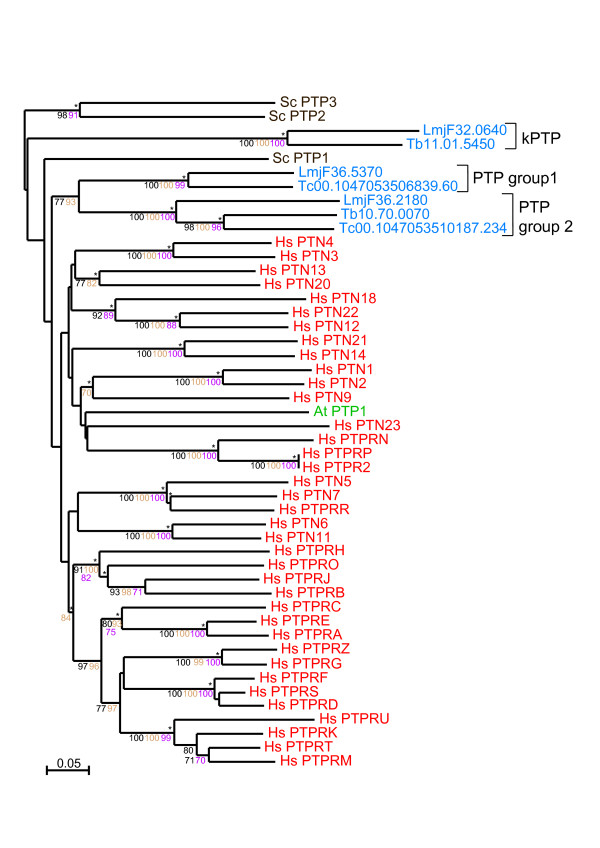
**Phylogram of TriTryp PTPs**. The phylogram of PTP catalytic domains includes TriTryp sequences and human, *S. cerevisiae *and *A. thaliana *as markers. Phosphatase domains are indicated by systematic gene IDs. Sequences are colour-coded by organism: blue for *T. cruzi* (*Tc*), *T. brucei *(*Tb*) and *L. major* (*Lmj*F); red for human (*Hs*); brown for *S. cerevisiae *(*Sc*) and green for *A. thaliana *(*At*). Protein names replace Swiss-Prot IDs for some human, yeast and plant sequences. Results of the four phylogenetic methods are shown: bootstrap values > 70 are black for Neighbour-Joining, brown for Bayesian and purple for Maximum Parsimony. Asterisks (*) show Maximum Likelihood support.

PTPs contain single polypeptide chains that form the catalytic domain and they are usually decorated with accessory subdomains (for example, SH2, Rhodanese, Ig, FN) critical for specific regulation or subcellular location [25,29]. PTPs are recognised by a highly conserved active-site motif, CX_5_R, necessary for a Cys-based mechanism of catalysis, assisted by a conserved Asp residue. The rest of the catalytic domain differs significantly between subfamilies. In addition to the classical PTPs a number of atypical phosphatases exist that lack catalytic activity (STYX or pseudophosphatases) [30]. All TriTryp PTPs identified have a single PTP domain (Figure 2) with the conserved active-site motif, CX_5_R, but with no extracellular regions or trans-membrane regions predicted, and without any additional recognisable regulatory or targeting domains commonly found in human PTPs. A sequence analysis shows that kinetoplastid PTPs fall into three separate groups (see Additional File 2) based on the conservation of the 10 landmark motifs known to be important for catalysis, substrate binding and maintenance of the three-dimensional fold characteristic of PTPs [25]. Group 1 contains the sequences that are the most similar to human phosphatase domains. A member of this group is present in *L. major *(*Lm*PTP1) with an orthologous syntenic gene in *T. cruzi *(*Tc*PTP1) (see Additional file 3). However, *T. brucei *lacks an orthologue of this protein, suggesting that it may have a role in intracellular parasitism. This is consistent with recent functional analysis of LmPTP1 demonstrating reduced virulence of amastigote forms upon genetic ablation [19]. Group 2 contains three proteins, *Tb*PTP1, *Lm*PTP2 and *Tc*PTP2 (Note that, despite the nomenclature, *Tb*PTP1 is not the orthologue of *Tc*PTP1 and *Lm*PTP1). We have recently characterised *Tb*PTP1 as a tyrosine specific PTP with a critical role in controlling *T. brucei *differentiation [18]. These Group 2 PTPs lack motif 2 (DX_2_RVXL) in the phosphatase domain and contain up to six kinetoplastid-specific regions in both the pre-catalytic and catalytic domain of the protein. Distinct specific motifs are also found in Group 1 PTPs with slight sequence variations (see Additional file 4). The function of these regions is unknown but may be potentially important in substrate recognition or regulation. Group 3 (kinetoplastid-specific PTPs, kPTPs) sequences show the most interesting variations of the PTP domain with substitutions in most motifs and a deletion between motifs 7 and 8. Substitutions were detected in the structural motifs (motifs 2–7) of five hydrophobic residues-required for core stability-, by hydrophilic and basic residues. Altogether, these changes may have a considerable effect on the stability of the PTP domain and perhaps this is compensated by alternative folding arrangements or local conformational adjustments. These may become clear once structural information on these enzymes is available. Phylogenetic analysis of the PTP sequences (Figure 3) confirms the presence of three clades, which are distantly related to human, *S. cerevisiae *and *A. thaliana *PTPs.

Because the analysis of the TriTryp kinome revealed that these organisms do not have tyrosine kinases, determining the exact roles of the uncharacterised PTPs has become more important, given their potential role in the regulation of parasite biology. Our analysis did not identify any potential receptor PTPs, which is consistent with the lack of receptor tyrosine kinases. The sequence differences described above and the low homology to human PTPs suggest that these phosphatases may be suitable targets for the design of specific and selective inhibitors against parasitic infection and transmission.

### Dual-specificity phosphatases

**Figure 4:**
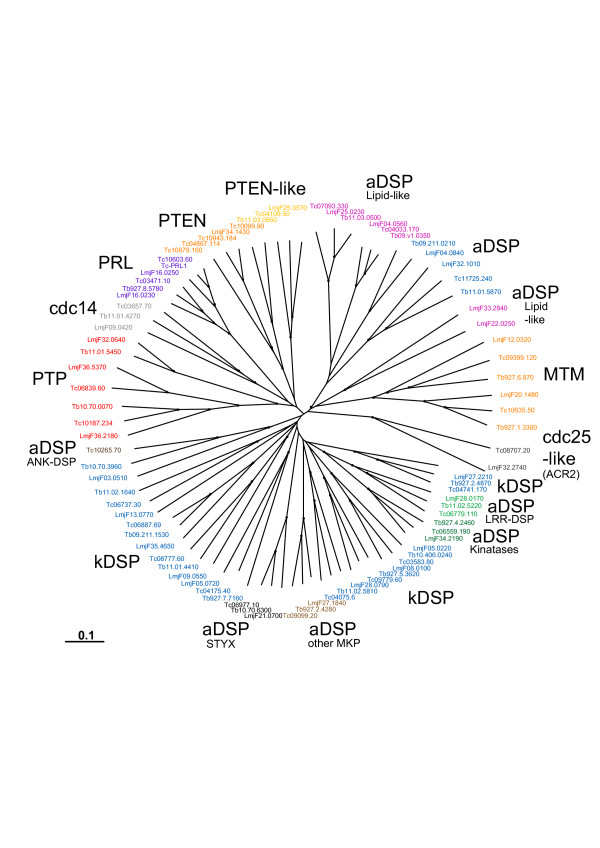
**Radial phylogenetic tree of TriTryp DSPs and PTPs**. Neighbour joining tree showing *T. cruzi *(*Tc*), *T. brucei *(*Tb*) and *L. major *(*Lmj*F) sequences. Sequence IDs are colour-coded according to groupings as labelled in the figure. Bootstrapping values > 70 are shown as dots on the branches. *T. cruzi *sequence IDs are truncated to the unique part, so the invariant 00.10470535 has been removed.

TriTryp DSPs show a large variety of domain architectures, some of them completely novel when compared to DSPs in other organisms (Figure 2). Sequence analysis of the type 2 DSP domains in the TriTryp genomes shows a group of parasite proteins with clear matches to classic eukaryotic DSPs (eDSPs). These sequences show good conservation of the five classic motifs in DSPs [24]. The eDSP group includes PRL (phosphatase of regenerating liver) and its isoforms, and cdc14 (cell division cycle 14) (Table 1, Figure 4).

#### eDSPs: cdc14 and PRLs

PRL phosphatases (PRL1, -2, -3) play important roles in cell growth and cellular transformation, potentially through regulation of spindle dynamics [31,32]. They show sequence similarity to cdc14, a DSP that regulates mitotic exit [33,34] and PTEN, a tumour suppressor [35], but they posses a unique C-terminal prenylation site or CAAX sequence, essential for localisation to the plasma membrane and early endosomes [36]. PRL phosphatases are found in the three kinetoplastids and all of them contain prenylation C-terminal CAAX sequences. PRL-1 in *T. cruzi*, has been characterised and it was found to be farnesylated at the C-terminus and to localise to endocytic membranes [37]. The functional role of this protein in trypanosomes is still unclear.

### Atypical DSPs (aDSPs)

The majority of TriTryp DSPs show significant differences to classic DSPs, and have either unusual domain organisations (Figure 2), or are lacking conservation of sequence features in their catalytic domain (see Additional file 5), we refer to this group as atypical DSPs (aDSPs). In the first category, we found three subfamilies of phosphatases with unusual domain organisation: 1) a catalytic DSP domain with additional Leucine Rich Repeats (LRRs) (types 4 and 5 in Figure 2); 2) a DSP domain with two pseudokinase domains (see below) and LRRs (types 6 and 7, Figure 2), we named this subfamily *kinatases *(from kinase and phosphatase); and 3) a DSP domain with an ankyrin domain. The presence of LRRs in a phosphatase sequence is very unusual. Related examples are only found in *A. thaliana*, which contains a large family of receptor protein kinases with LRRs [38] and also in some human kinases, for example, LRRK1 [39]. The only other example of a phosphatase with LRRs is the human protein PHLPP, which dephosphorylates Akt and promotes apoptosis [40]. This protein contains LRRs with a protein phosphatase 2C domain. No sequences have been reported to date containing both LRRs and DSP domains or kinase domains with LRRs and DSP domains, making these domain organisations unique to the kinetoplastids.

The LRR regions from each of the kinetoplastid sequences were investigated using sequence similarity searching. Interestingly, the LRRs in the kinatase group have homology to LRR modules in SHOC2/SUR-8 [41]. The LRR-DSPs match SHOC-2, or Soc-2 proteins, but the E-values for all the matches are much higher (>0.001). SHOC2 is a Ras-binding scaffold protein that enhances Ras-MAP kinase signal transduction by facilitating the interaction between Ras and Raf [42]. Other matches include a human sequence (Q9Y4C4_HUMAN, TrEMBL database) annotated as "Malignant fibrous histiocytoma amplified sequence", which contains a Ras GTPase domain, and plant intracellular Ras-group related LRR proteins (PIRLs) [43], related to Ras-binding proteins in animals and yeast. All three kinatases and all three LRR-DSPs also have near-top matches to bacterial LRR-containing sequences annotated as small GTP-binding proteins (e.g. *Trichodesmium erythraeum *Q10Y31_TRIEI, *Magnetococcus *A0L4U3_MAGSM, and *Anabaena variabilis *Q3MD20_ANAVT). Overall, the kinatases and LRR-DSPs show similarity to LRR proteins involved in regulation of Ras-mediated signalling pathways and small GTP-binding proteins in bacteria, suggesting that they may share similar functional roles in the parasites and act as scaffolding proteins in signalling pathways.

**Figure 5:**
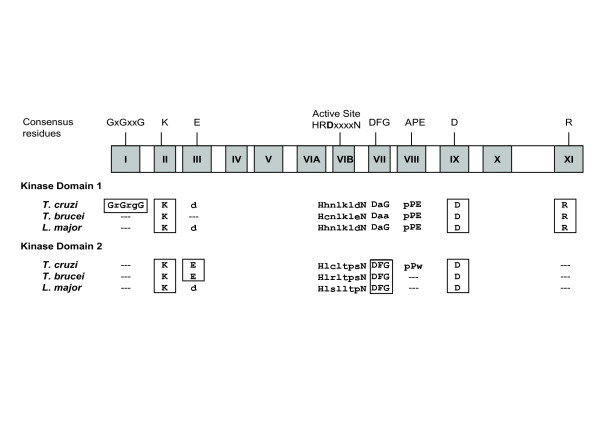
**Conservation of protein kinase motifs in the kinetoplastid 'kinatases'**. The 11 subdomains of eukaryotic protein kinases are represented as blocks, with essential conserved residues for catalysis marked above. Analysis of the both kinase domains from the three kinatases is shown underneath. Fully conserved motifs are boxed in black and conserved residues from partially conserved regions are in bold type.

Analysis of the kinase domains in the kinatases indicates that they are likely to be enzymatically inactive, and we refer to them as pseudokinases for consistency with the previous classification. Comparison to eukaryotic protein kinases highlights the lack of residues essential for catalytic activity and substrate binding (Figure 5). For example, the glycine triad (GXGXXG) necessary for ATP binding is only present in one of the *T. cruzi *kinase domains, and the catalytic Asp residue in the active site HRD motif is substituted by other residues in all of the kinase domains. The first kinase domain in the kinatases also has substitutions in the magnesium-binding DFG motif. Although these pseudokinase domains might have lost the capability to phosphorylate substrates, they may still perform important regulatory roles in signalling pathways as has been reported for other pseudokinases [44]. The presence of the LRR modules strongly suggests a role in protein-protein interactions, while the addition of an active DSP domain and pseudokinase domains hints to a sophisticated regulatory function, which has not been reported in higher eukaryotes. A similar domain architecture (kinase + phosphatase) is present in other protozoan organisms such as *Giardia lamblia*, *Tetrahymena thermophila*, and *Dictyostelium discoideum*, indicating evolutionary conservation of a these proteins. Sequences with LRRs and DSP domains only, are also present in other protozoa: *Entamoeba histolytica *and *Dictyostelium discoideum *suggesting that this family may be also specific to protozoa. It will be interesting to further explore the functional relevance of these novel DSPs in kinetoplastid signalling.

The last type of atypical domain organisation is found in *T. cruzi *and contains a DSP domain with an additional ankyrin domain (type 8, Figure 2). Ankyrin is a common protein-protein interaction domain found in proteins involved in transcription initiation, cell cycle regulation and signalling [45]. There are few examples of PTPs with Ankyrin domains. One of these is the human protein tyrosine phosphatase PTN20 variant 11 (Q4JDK8_HUMAN). Others include uncharacterised sequences from *C. elegans *and *C. briggsae *(Q22668_CAEEL and Q628I2_CAEBR). No DSPs have been reported with ankyrin domains thus this appears to be a novel domain architecture only present in *T. cruzi*.

In a second category of aDSPs, we found a number of sequences that are particularly different from eukaryotic DSPs with low homology in one or more functional DSP motifs, and with amino acid substitutions in the active site P-loop (see Additional file 5). These sequences can be grouped into 6 further subgroups. Groups 1, 2 and 3 contain basic residues Arg or Lys in the P-loop signature, a trait found in PTEN lipid phosphatases, although in the phylogenetic tree they cluster independently of PTEN. We refer to them as lipid-like aDSPs (Figure 4 and Table 1). Group 3 have similar features in the P-loop to the triple-specificity phosphatase MptpB in *M. tuberculosis *[46]. Groups 4 and 5 lack the catalytic Arg in the P-loop and are missing either the D-motif or the R-motif. This group clusters around the lipid-like DSPs (aDSP in Figure 4). Another group (group 6 in Additional file 5) has substitutions of the catalytic Cys residue in the P-loop, a diagnostic of inactive phosphatases, also called STYX phosphatases. STYX phosphatases are evolutionarily conserved pseudophosphatase modules [30] that, like pseudokinases are enzymatically inactive, but are functionally important as modulators of phosphorylation-dependent signalling. Their actions may be implicated in either competitive or cooperative mechanisms of phosphosubstrate binding and controlling subcellular location as reported for STYX and inactive myotubularins [47,48] and the inactive phosphatase domains of receptor PTPs [49].

### MKPs

**Figure 6:**
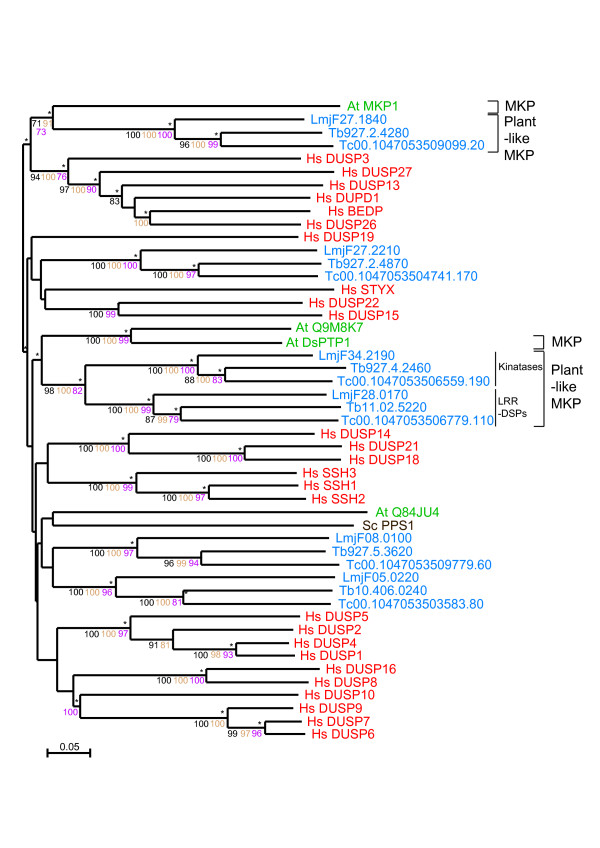
**Phylogram of TriTryp MKPs**. The phylogram of MKPs shows DSP catalytic domains from TriTryp sequences and human, *S. cerevisiae *and *A. thaliana *as markers. Phosphatase domains are indicated by systematic gene IDs. Sequences are colour-coded by organism: blue for *T. cruzi* (*Tc*), *T. brucei *(*Tb*) and *L. major* (*Lmj*F); red for human (*Hs*); brown for *S. cerevisiae *(*Sc*) and green for *A. thaliana *(*At*). Protein names replace Swiss-Prot IDs for some human, yeast and plant sequences. The results of the four phylogenetic methods are shown: bootstrap values > 70 are black for Neighbour-Joining, brown for Bayesian and purple for Maximum Parsimony. Asterisks (*) show Maximum Likelihood support.

MAP kinase phosphatases (MKPs) are critical regulatory proteins in stress-dependent and hormone-related signalling pathways, and are highly conserved in vertebrates. However, other eukaryotes, like yeast and plants, have distinct MKPs apparently unrelated to the vertebrate analogues [50-53]. There are no obvious orthologues of human MKPs in kinetoplastids. Instead, a number of sequences were found to have close homology to reported MKP phosphatases in *Arabidopsis *(*At*MKP1, *At*DSPTP1). This is supported both by blast searches and clustering in the phylogenetic tree (Figure 6). These sequences segregate into two groups in the tree (Figure 6), one of them includes the atypical DSPs with LRR motifs and the kinatases. The other group clusters with AtMKP1. One sequence in the second group, Tc00.1047053509099.20, has a KIM-like motif (kinase interacting motif) [54], although it does not contain the remaining motifs, found in classic MKPs [53] and shared with different MAP kinase (MAPK) effectors [55], or the rhodanese domain found in human MKPs. Furthermore, the close resemblance to plant MKPs highlights their divergence from the mammalian genes. The interesting combination with protein interacting modules, like LRR (discussed above) and pseudokinase domains, suggests a role in the regulation of signalling pathways, likely of the Ras-dependant MAPK type. There are a total of 42 protein kinases in the *T. brucei, T. cruzi *and *L. major*, which are thought to be regulated as MAPKs [28] and it is possible that the aDSP-MKPs identified here play a role in their regulation.

### Kinetoplastid-specific DSPs (kDSP)

A significant number of sequences in the DSP family share most of the classic DSP motifs, although they have no clear homology to any reported DSP and so they appear to be kinetoplastid-specific (Table 1, Figure 4). To determine kDSP function, further experimental characterisation will be needed.

### Special features and accessory domains

A feature of the kinetoplastid DSPs is that most of them, including the eDSPs, are significantly longer than human DSPs. The same is true for several kinases, in particular MAPKs [56,57]. A calculation of the average human DSP length gave 392aa whereas the figures for the kinetoplastids were: *T. cruzi*, 501aa; *T. brucei*, 519aa; and *L. major*, 700aa. Many of the parasite DSPs, have either N- or C- terminal extensions, some of them with no significant matches (E-value < 0.01) to annotated proteins such that no functions for these extra regions can be assigned. Other extensions contain accessory motifs or domains including the LRRs, pseudokinase domains and ankyrin domains previously described. Another example is the presence of a CAAX box, consisting of a cysteine (C), two aliphatic residues (A, A) and any other amino acid (X) in the PRL phosphatases. The cysteine directs protein prenylation important for localisation of the PRLs.

In contrast, some TriTryp DSPs, lack typical accessory modules found in mammalian genes. Such is the case of the putative MKPs that lack the kinase-binding rhodanese domain or CH2 domain [58] in the N-terminal region. This is also missing in mammalian low molecular weight MKPs, although they are still capable of dephosphorylating MAPKs. In the kinetoplastid MKPs the presence of LRRs and pseudokinase domains may replace the specific binding role of such domains. Thus, the kinetoplastid PTPs and DSPs seem to have adopted different domain organisations and strategies to fulfil analogous functions to mammalian phosphatases. A similar trend was also observed for protein kinases, which in trypanosomes lack the same type of accessory domains as their human orthologues. In fact, four of the five most common Pfam domains in human protein kinases are absent in the trypanosomatids kinome [28].

### Lipid phosphatases (PTEN and myotubularins)

**Figure 7:**
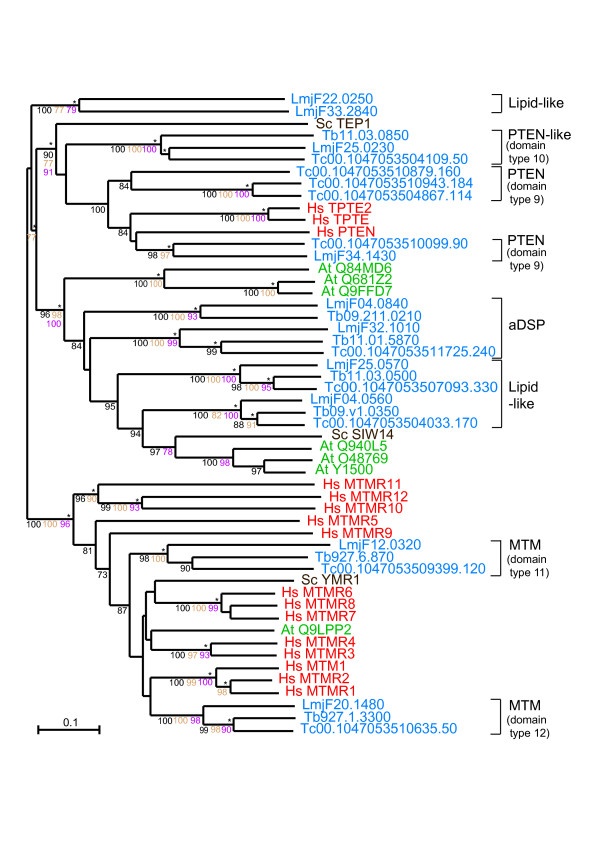
**Phylogram of TriTryp lipid phosphatases**. The phylogram of lipid phosphatases shows DSP/lipid catalytic domains from TriTryp sequences and human, *S. cerevisiae *and *A. thaliana *as markers. Phosphatase domains are indicated by systematic gene IDs. Sequences are colour-coded by organism: blue for *T. cruzi* (*Tc*), *T. brucei* (*Tb*) and *L. major* (*Lmj*F); red for human (*Hs*); brown for *S. cerevisiae *(*Sc*) and green for *A. thaliana *(*At*). Protein names replace Swiss-Prot IDs for some human, yeast and plant sequences. The results of the four phylogenetic methods are shown: bootstrap values > 70 are black for Neighbour-Joining, brown for Bayesian and purple for Maximum Parsimony. Asterisks (*) show Maximum Likelihood support.

PTEN and myotubularins (MTMs) are DSP phosphatases with activity against phosphoinositides. This peculiar substrate versatility is crucial to their identified biological roles in mammals as tumour suppressors (for PTEN), and as regulators of endosomal trafficking (in the case of MTMs). Their role in lipid dephosphorylation and membrane targeting may also be important in different aspects of kinetoplastid metabolism. PTEN-like phosphatases were identified in the three kinetoplastids (Table 1) with two different architectures: the first has a DSP domain and a calcium-binding region at its C-terminus (type 9, Figure 2) and the second contains only the DSP domain (type 10, Figure 2) and shows lower homology to eukaryotic PTENs. The existence of two types of PTEN phosphatases is also reflected in the phylogenetic analysis, even when the DSP domain alone is considered (Figure 7). The type 9 group is closest to human PTEN and contains 4 *T. cruzi *and 1 *L. major *sequence (Figure 7). Interestingly, *T. brucei *does not seem to have an orthologue of human PTEN, but it has a PTEN-like protein similar to the other two parasites.

Two subfamilies of MTMs were identified that possess the typical domain organisation of MTMs, with a phosphatase domain and N-terminal lipid-binding GRAM domain (type 11, Figure 2), or with and additional PI_3_P binding FYVE domain (type 12, Figure 2). Each of the parasites has one sequence of each type. Overall, the six kinetoplastid MTM sequences have high similarity to human MTMs across the length of the phosphatase domain. However, they do not individually cluster with any human MTMs, hence direct orthologues cannot be determined (Figure 7). It is interesting that only two MTM sequences are present in each of these organisms as this is one of the largest subfamilies of DSPs in humans (14 genes). Along with the classical types of DSPs, the MTM family appears to have been substantially expanded in higher eukaryotes. MTMs, which are relatively large sequences in humans, are larger still in the kinetoplastids. Most human MTMs are around 450–700aa with a few over 1,000aa. The smallest TriTryp MTM is 872aa (Tb927.1.3300) and the largest is 3,246 aa (LmjF12.0320) with a long N-terminus extension that has no predicted domain or motif matches.

### Cdc25 and Arc2 reductases

Cdc25 (cell division cycle 25) phosphatases are widely spread in metazoans and known to be responsible for the activation of cyclin dependent kinases (CDKs) [59]. This is an essential step in progression to mitosis that controls the G2 to M checkpoint. The kinetoplastid cell cycle is also regulated by CDK-related kinases (CRKs) [60] and 10–11 CRKs have been identified in the TriTryp kinome [28]. Although modulation of CRK activity is still not well understood, the presence of other cell cycle regulatory phosphatases like PP1, PP2A and cdc14 suggest that similar mechanisms may be conserved in kinetoplastids.

In our analysis, two sequences, Tc00.1047053508707.20 and LmjF32.2740, matched the rhodanese-like domain similar to the cdc25 phosphatase catalytic domain. No significant match was found in *T. brucei *(< 0.01). Orthologues of LmjF32.2740 are also present in *L. infantum *and *L. brazilensis *(see Additional file 6).

Rhodanese domains are also found in sulfur-transferases and in the Acr2 type of arsenate reductases, indicating a common evolutionary lineage with phosphatases [61,62]. In addition, they all share a similar Cys-based catalytic mechanism and a conserved CX_5_R active site motif [61]. In fact, LmjF32.2740 is homologous to eukaryotic arsenate reductases from *S. cerevisiae*, *Sc*ACR2, and *Arabidopsis*, *At*ACR2 (formerly annotated as cdc25). Consistent with this, LmjF32.2740 (*Lm*ACR2) has been found to functionally complement the arsenate-sensitive phenotype of the *Sc*ACR2 deletion in *S. cerevisiae*. Furthermore, it confers sensitivity to the antimonial drug Pentostam, by virtue of its antimonial reductase activity in addition to the arsenate reductase activity [63]. Recently, it has been reported that purified *Lm*ACR2 also exhibits apparent phosphatase activity in vitro [64]. Tc00.1047053508707.20 remains uncharacterised, but its similarity to *Lm*ACR2 predicts it may have arsenate reductase activity. Phylogenetic analysis further supports the idea that cdc25-like sequences in *Leishmania *and *T. cruzi*, are more closely related to eukaryotic Acr2 reductases than to mammalian cdc25s (see Additional file 6). The active site motif of the kinetoplastid sequences is clearly different in composition and properties to the mammalian cdc25 that have acidic residues (EFSSE), but similar to Acr2s with hydrophobic residues (AXSLV).

The functional role of cdc25 in kinetoplastids, as suggested for plants [65], may be replaced instead by the activity of B type cyclin-dependent kinases (cdck-B). The nearest homologues to the cdck-B in *Arabidopsis *are the Cdc2-related kinases (CRK1–3, [66]) identified in *T. brucei *and *L. mexicana*. These CDKs are critically involved in cell cycle control mechanisms similarly to Cdc25s in other eukaryotes [67-69].

### LMW-PTPs and ArsC reductases

Three kinetoplastid sequences (Tb09.160.2100, LmjF01.0200 and Tc00.1047053504797.120) were initially found to match the InterPro LMW-PTP domain (IPR000106). These sequences were aligned with more than 250 LMW-PTPs from different organisms and also with a group of related bacterial arsenate reductases (ArsC type) for phylogenetic analysis (data not shown). The prokaryote ArsC reductases are unrelated to eukaryote Acr2 reductases, and yet they also share the active site P-loop signature motif CX_5_R with PTPs and have remarkable structural similarity to LMW-PTPs [70]. The kinetoplastid sequences formed a separate cluster in the phylogenetic tree originating from the same branch as those of the bacterial ArsC but clearly distinct from the LMW-PTPs. The phylogram (see Additional file 6) also shows that cdc25 and Acr2 (yeast, plants and kinetoplastids) are clearly related but distinct from the ArsC reductases found in bacteria and in kinetoplastids.

The three kinetoplastid ArsC sequences have two conserved long insertions. Interestingly, the kinetoplastid ArsC sequences lack the catalytic Cys residue in the active site (substituted by Gly), and the two landmark Cys residues also important in catalysis [71,72]. However, they contain the catalytic Arg (P-loop) and Asp (general acid in catalysis) residues. This suggests that they may be inactive enzymes or that they use an alternative catalytic mechanism.

ArsC homologues are not present in vertebrates or plants, which use Acr2 instead for metalloid detoxification. It is intriguing then that *T. cruzi *and *L. major *have retained the *bacterial *arsenate reductases ArsC in addition to acquiring the eukaryotic Acr2 and that the *Acr2 *gene is not present in *T. brucei*, – with the syntenic region of the genome in that parasite instead containing two predicted amino acid transporter genes (see Additional file 7). Recently, the presence of two putative ArsC proteins in *A. fumigatus *has been reported [73], suggesting that some lower eukaryotes may require both systems for arsenate reduction.

### Serine/threonine phosphatases

STPs are multi-subunit complexes, which combine a catalytic subunit with various regulatory and targeting subunits [26,74] providing selective substrate specificity, subcellular localisation and regulation of enzymatic activity. STPs share a similar catalytic mechanism and are structurally related, although based on metal ion dependence and sequence homology they can be divided into three main subfamilies: PPP, PPM and FCP [26]. The PPP and PPM subfamilies share structural similarity and a common mechanism of catalysis, with conserved acidic residues that coordinate the metal ions essential for activity. In humans, a small number of genes encode for STP catalytic domains. Conversely, in *C. elegans *[75] and *Arabidopsis *[76], STPs represent between 50–80% of the phosphatome. We restricted our analysis to the catalytic phosphatase domains for this class. The STP family of phosphatases is the largest represented in kinetoplastids accounting for more than one half of the whole complement of protein phosphatases. Typical domain architectures were identified (Figure 2) containing the conserved phosphatase catalytic domain either alone or with accessory domains like tetratricopeptide repeats (TPRs) that mediate protein-protein interactions, found in PP5 or calcium binding EF-hand domains, found in PPEF proteins. Many kinetoplastid STPs, like the DSPs, are longer proteins than their human relatives, with extensions of unknown function.

### PPP phosphatases

PPP phosphatases PP1 and PP2A are involved in the regulation of a number of signalling pathways, including MAPK-dependent networks, cell cycle regulation, glycogen metabolism and microtubule organisation at centrosomes [77-81]. The general pattern that identifies members of the PPP family contains motifs from three separate regions G**D**X**H**G – G**D**XVDRG – G**N**HE [82] (residues in bold coordinate metal ions at the catalytic site and the underlined His is the proton donor in catalysis).

**Figure 8:**
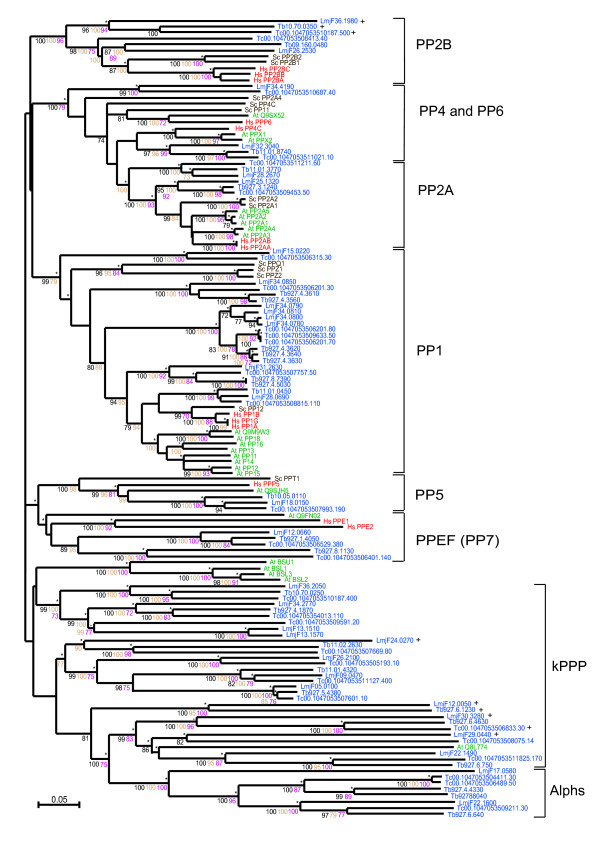
**Phylogram of the PPP subfamily of serine/threonine phosphatases**. Included are TriTryp PPP catalytic domains and those from human, *S. cerevisiae* and *A. thaliana*. Phosphatase domains are indicated by systematic gene IDs. Sequences are colour-coded by organism: blue for *T. cruzi *(Tc), *T. brucei* (Tb) and *L. major* (LmjF); red for human (Hs); brown for S. cerevisiae (Sc) and green for A. thaliana (At). Protein names replace Swiss-Prot IDs for some human, yeast and plant sequences and systematic IDs for the parasites. Results of the four phylogenetic methods are shown: bootstrap values > 70 are black for Neighbour-Joining, brown for Bayesian and purple for Maximum Parsimony. Asterisks (*) show Maximum Likelihood support. The symbol '+' marks kinetoplastid sequences with catalytic mutations (listed in Additional file 8).

There are clearly identifiable clades in the PPP phylogenetic tree for the PP1, PP2A, PP2B, PP4, PP5 and PPEF phosphatases and these groups are well supported statistically (Figure 8).

### Protein phosphatase 1 (PP1)

The PP1 group contains a large group of kinetoplastid sequences: 7 in *T. cruzi*, 8 in *T. brucei *and 8 in *L. major*. Two of the *T. cruzi *PP1catalytic subunits (PP1α and PP1β) have been characterised [20]. Inhibitor studies suggested that in *T. cruzi *epimastigotes, PP1 has an important role in the completion of cell division and in the maintenance of cell shape [20]. Similar roles were previously proposed for PP1 in higher eukaryotes [83]. The functional roles of the *T brucei *PP1 and PP2A phosphatases have also been studied [84]. The combined RNAi ablation of 7 PP1s and the PP2A catalytic subunits in procyclic forms, resulted in a slow growth phenotype but did not lead to lethality. This is in contrast with previous experiments in which trypanosomes treated with okadaic acid (OA), a well known inhibitor of PP1 and PP2A enzyme activity, show defects in cytokinesis and organelle genome segregation (multinuclear cells with single kinetoplast) [85]. The seemingly conflicting findings of these two reports (absence of multinucleated cells with RNAi vs. multinucleated cells after okadaic acid treatment) suggests that additional OA sensitive PPP enzyme activities are present in *T. brucei *although incomplete ablation by RNAi might also be responsible.

Interestingly, 3 PP1c genes in *T. cruzi*, 4 PP1c genes in *T. brucei *and 5 PP1c genes in *L. major *are found in tandem gene arrays. In *T. brucei *the repeat structure of the array is disrupted through extensive divergence in the flanking and coding sequences. The gene encoding PP1-7 (Tb927.4.3560) has evolved unique UTRs and is separated from the other 3 members (PP1-6 (Tb927.4.3620), PP1-5 (Tb927.4.3630) and PP1-4 (Tb927.4.3640)) by the insertion of two unrelated tandem-arranged genes (each encoding translational elongation factor 1 beta). This organisation retains orthology in both *T. cruzi *and *L. major *[86]. Kinetoplastid genes do not generally possess individual promoters and, therefore, cannot be up-regulated through transcriptional initiation. Instead, where high transcript levels are required, genes may be duplicated to form tandem gene arrays, which are co-transcribed. The PP1s in the arrays might needed to be expressed on a certain level, working as "housekeeping genes" while the other PP1 homologues would be in charge of the "fine tuning" of various signal transduction events.

### Protein phosphatase 2A and 2B (PP2A and PP2B)

Two groups of PP2A phosphatases were identified for each parasite. One group is more closely related to human, *S. cerevisiae *and plant PP2As than the other (Figure 8). A member of the second group, PP2A from *T. cruzi *(Tc00.1047053511021.10), has already been characterised as important for the complete transformation of trypomastigotes into amastigotes during the life cycle of the parasite [87]. Similarly to PP2A, two groups of PP2B are present in kinetoplastids. One group clusters with the human and *S. cerevisiae *homologues, while the second triplet forms a separate cluster (Figure 8). Sequences in the second cluster have mutations in catalytic residues suggesting they may be pseudophosphatases.

### Protein phosphatase 4 and 6 (PP4 and PP6)

Homologues to human PP4 and PP6 have not yet been characterised in any of these kinetoplastids. We found a well-supported PP4 cluster that included a kinetoplastid triplet together with the human and *A. thaliana *PP4 sequences (Figure 8). PP6 orthologues were found in *T. cruzi *(Tc00.1047053510687.40) and *L. major *(LmjF34.4190) by blast search, but there is apparently no *T. brucei *PP6 orthologue.

### Protein phosphatase 5 (PP5)

Homologues of PP5 in *T. brucei, T. cruzi *and *L. major *cluster with human and *S. cerevisiae *PP5 sequences (Figure 8) and they all contain tetratricopeptide (TPR) modules (Figure 2). Analysis of the *T. brucei *PP5 (*Tb*PP5) [22] highlights that the TPRs of this protein are actually similar to those in fungi. *Tb*PP5 expression is regulated during cell cycle progression and it is important for normal cell growth [88].

### Protein phosphatases with EF Hands (PPEF)

Close to the PP5 sequences are the protein phosphatases with EF-hands (PPEF) (or PP7) (Figure 8). Kinetoplastid PPEF phosphatases are found clustering with human PPEFs, showing that there are two PPEF proteins in *T. cruzi *and *T. brucei *but only one in *L. major*. As there are two human PPEF proteins this suggests either that duplication of the PPEF gene occurred before kinetoplastids diverged and that *L. major *has lost a copy or that two duplication events occurred independently, in kinetoplastids and during higher eukaryote development. A recent publication discusses the PPEF family of kinetoplastid phosphatases [23], showing that these enzymes are N-myristoylated and constitutively expressed through all parasites life cycle.

### Other PPPs

In many eukaryotic organisms there exist "non-conventional" PPPs. These have greater similarity to bacterial enzymes than to other eukaryotic PPP proteins [89]. Alphs (ApaH-like phosphatases) are a group of eukaryotic PPPs that have greater similarity to bacterial diadenosine tetraphosphatases than to other members of the PPP family and several kinetoplastid PPP sequences fall into this category (see Additional file 8). These Alphs have specific mutations in the second conserved motif, GDXVDRG, in particular, the substitution of the second Asp for a neutral residue and substitution of the Arg for Lys [89]. These mutations are found in 3 *T. cruzi*, 3 *T. brucei *and 2 *L. major *sequences (Additional file 8).

### Kinetoplastid-specific PPPs (kPPP)

Several kinetoplastid PPP sequences have amino acid substitutions of catalytically important residues (Additional file 8). These pseudophosphatases may not be able to carry out dephosphorylation, although they may still have kinetoplastid-specific roles. These sequences form part of a large group of kinetoplastid-specific PPPs (kPPP, Figure 8). The long branch-lengths of several of the proteins in this group and also the Alphs indicate that these kPPP sequences have diverged much more over time than the others. The most interesting aspect of the kPPP group is that sequence similarity searches show some of these sequences have greater similarity to plant and fungal phosphatases, in particular to the BSU1 and BSL types of *A. thaliana *phosphatases. BSU1 is a nuclear protein phosphatase that modulates the cell response to plant steroid hormones [90]. One of these kinetoplastid-specific sequences, Tb927.6.4630, has previously been identified as a "Shelph" with similarity to PPP phosphatases from Shewanella, a psychrophillic bacteria [89]. Another Shelph exists in *L. major*, LmjF31.2630 that appears in the PP1 section of the tree (Figure 8).

### PPM phosphatases

Despite differences in their primary sequences, the three-dimensional structures of PPP and PPM proteins are very similar and they share a common catalytic mechanism [91]. PPM phosphatases depend on Mg^2+ ^or Mn^2+ ^for catalytic activity. A set of 11 conserved motifs has been identified within the PPM/PP2C family of phosphatases [92]. Motifs 1, 2, 5, 6, 8 and 11 are most conserved in eukaryotes and form the pattern: **ED **– **DG**H [AG] – GD – GD – **DG **– **D**N (conserved Asp residues coordinate the metal ions Mg^2+ ^or Mn^2+ ^essential for catalysis). The analysis of the PP2C sequences from kinetoplastidsshows that all of them possess the Asp residues in the motifs above, with the only exception of Tc00.1047053504163.10, Tb10.70.1410 and LmjF36.1260, which are missing some of the acidic residues in the metal binding site. These have been previously designated as 'PP2C-like' [2] because they do not possess all of the PP2C motifs.

**Figure 9:**
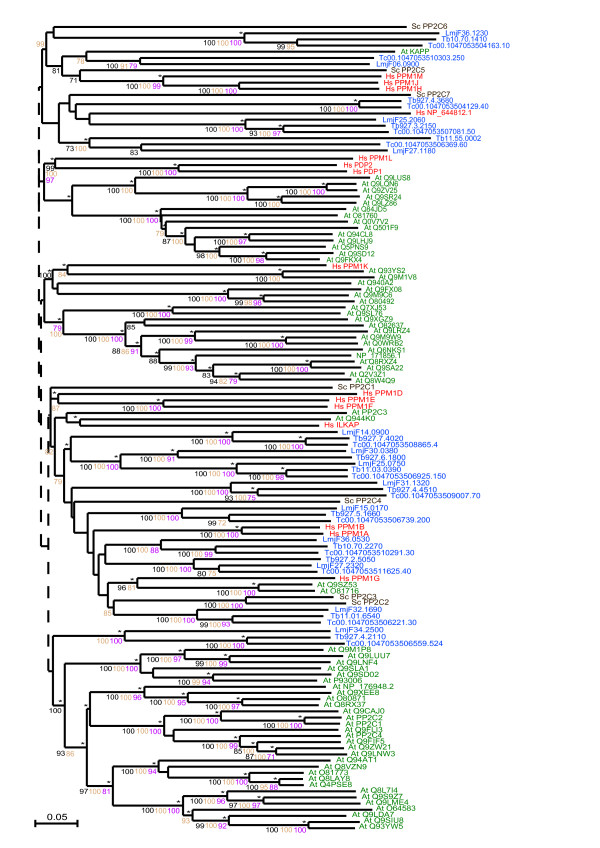
**Phylogram of the PPM type of serine/threonine phosphatases**. This phylogram includes PPM catalytic domains from human, *S. cerevisiae *and *A. thaliana*. Phosphatase domains are indicated by systematic gene IDs. Sequences are colour-coded by organism: blue for *T. cruzi* (*Tc*), *T. brucei *(*Tb*) and *L. major* (*Lmj*F); red for human (*Hs*); brown for *S. cerevisiae *(*Sc*) and green for *A. thaliana *(*At*). Most sequence IDs are from the Swiss-Prot database but there are also NCBI database accession numbers used (beginning 'NP'). Results of the four phylogenetic methods are shown: bootstrap values > 70 are black for Neighbour-Joining, brown for Bayesian and purple for Maximum Parsimony. Asterisks (*) show Maximum Likelihood support. Dashed lines show phylogenetic relationships as indicated in an initial tree from an ungapped alignment. Each clade was analysed separately to obtain robust phylogenetic analysis and these were then combined to show the whole PPM family.

The TriTryp PPM family is similar to the human as there are 15 PPMs in human and 14 in *T. cruzi*, 13 in *T. brucei *and 15 in *L. major*. *A. thaliana*, however, has a larger expansion of this family [76] with 63 genes. From the phylogenetic analysis (Figure 9) we observed that plant PPM phosphatases, with few exceptions, form distinct clades separate from human, yeast and kinetoplastid sequences. Parasite PPMs have greater similarity to human and yeast sequences, although direct human homologues are not clear from the tree.

PP2C phosphatases are negative regulators of stress-regulated signalling mediated by PKA and the p38 and JNK MAPK pathways [93,94] in mammals. In yeast and plants, PP2C proteins are also involved in modulating stress response signalling [95-97]. More recently an important role in cell cycle regulation has been reported for human PP2C in dephosphorylation of Cdk2 and Cdk6 [98], and in cell cycle arrest in *Xenopus *[99]. Other functions of PP2Cs include the regulation of cytoskeleton organisation [100] and development [101]. A *Leishmania *PP2C, *Lc*PP2C, was shown to be present in both the infective promastigote and tissue amastigote stages of *L. chagasi *and *L. amazonensis*. The catalytic properties of LcPP2C were found to be similar to eukaryotic PP2C enzymes with respect to Mg^2+ ^dependence and insensitivity to the inhibitor okadaic acid [102].

### FCP phosphatases

This family comprises a group of phosphatases that dephosphorylate the carboxy-terminal domain (CTD) of RNA polymerase II and that interact with transcription factor TFIIF [103,104]. In yeast, *FCP1 *is an essential gene [104]. We found FCP homologues in all three genomes: 13 in *T. cruzi*, 14 in *T. brucei *and 13 in *L. major *matching human and *S. cerevisiae *proteins (see Additional File 1). These phosphatases dephosphorylate serine residues of the conserved "heptad repeats" YSPTSPS at the CTD of the polymerase, which is required for recycling of the polymerase at the end of transcription. *T. brucei *has an RNA polymerase II, but this protein lacks the heptad repeats at its C-terminus, as found in most eukaryotes. Despite the lack of heptad repeats, the *T. brucei *RNA polymerase II is believed to be phosphorylated at alternative sites – perhaps through the C-terminal region which is rich in serine and tyrosine residues-, that may be targeted by the identified CTD phosphatase homologues [105,106].

Overall, there is a remarkable expansion of the kinetoplastid STP family in comparison to other eukaryotic genomes. Importantly, this highlights the prominent role of serine/threonine phosphorylation in the physiology of these parasites that overwrites the importance of tyrosine phosphorylation as reported in mammalian organisms. This is consistent with the lack of tyrosine kinases [28] and matches the situation in plants [76].

## Conclusion

The fluctuation of protein phosphorylation in kinetoplastids is well documented and in many instances is clearly related to stage-specific events or cell cycle regulation. As the functional characterisation of protein kinases is quickly expanding, a better knowledge of the phosphatase complement of these unicellular parasites is essential to understand the complexity and regulation of many cellular processes controlled by phosphorylation. Overall we identified more than 250 protein phosphatase domains in the three kinetoplastids, which represents about 50% of the kinase genes and apparently indicates a lower level of complexity for this type of enzymes. Further complexity may result from combinations with interacting regulatory subunits, particularly in the STP family or by stage-specific control of their expression levels.

The analysis of the TriTryp phosphatome provides valuable information for future experimental studies and highlights many interesting differences with other eukaryotic genomes, such as the low proportion of PTPs and the expansion of the STP family. Interestingly, a larger number of atypical protein phosphatases have been identified in these species, comprising approximately one third of the total. Most of the atypical phosphatases belong to the DSP family, and show considerable divergence from classic DSPs. Novel domain architectures suggest potential functional roles for the LRR containing DSPs as scaffolds in cellular events. Many kinetoplastid phosphatases are longer than human phosphatases and those extensions may contain unidentified functionalities. Sequence extensions have also been found in PP2 phosphatases in *Plasmodium falciparum *[107] and in a large number of proteins from pathogenic microorganisms [108]. It appears that the presence of such extensions has been conserved through evolution and may have an important role in pathogenesis.

Between the three kinetoplastids there are interesting differences, potentially related to their different environments and parasitic mode. *T. brucei*, the only extracellular pathogen, seems to have distinct traits, with less phosphatase genes than *T. cruzi *and *L. major*. Finally, the STP family appears to be extended in the TriTryp genome in comparison to humans, maybe to compensate for the lower number of PTPs. This correlates well with results from the TriTryp kinome study showing that these parasites are lacking tyrosine specific kinases. Overall, a number of important differences in the phosphatome of the TriTryp suggest that phosphorylation-dependent processes in these organisms may have a distinct organisation and physiological imprint that is worth a more detailed experimental exploration. Moreover, these distinct traits may be exploited in the selection of new targets for drug-design and development of therapeutic compounds taking advantage of the existing specific protein phosphatase inhibitors.

## Methods

### Ontology classification

The three protein data sets were obtained from GeneDB [109] (versions released in May 2006). The *T. cruzi *genome is a hybrid of two very closely related species and so many genes are present in two very similar forms. Using the Clusters of orthologous genes data [2] and sequence similarity searching, the shortest of each protein duplicate was removed. The phosphatase classification process was performed in a similar way to the method used previously to classify phosphatases from the human and *Aspergillus fumigatus *genomes [27]. Four components are required for the ontology classification: the phosphatase ontology (written in the Web Ontology Language OWL [110]), a description logic reasoner (Racer Pro version 1.9.0) [111], the Instance Store [112] and the domain compositions of all protein sequences, obtained through InterProScan [113], (version 13.1 of the InterPro database) [114]. The OWL ontology for the protein phosphatases was constructed using the Protégé ontology editor [79], which enables different types of protein phosphatase family members to be defined in terms of their domain architectures. These ontological descriptions of types of phosphatase were combined with the InterProScan domain information for individual proteins from each genome and the classification against the ontology was carried out through the Instance Store. The Instance Store combines a description logic reasoner and a relational database and provides an interface through which the data can be queried to return the results of the classification. The reasoner checks for logical inconsistencies in the ontology and performs the classification of individual phosphatases according to the types described in the ontology. The relational database of the Instance Store enables large numbers of protein instances to be stored. This system provides, in essence, an extension to the InterProScan domain-matching tool, as it is able to work at the level of a whole genome and place proteins in defined classes.

Using dual-specificity protein phosphatases (DSPs) as an example of the process, they are defined in the ontology as any protein possessing, amongst other features, a generic PTP domain (based on the HCX_5_R consensus motif) (IPR000387) and also a more specific DSP domain (IPR000340). When the Instance Store is queried for all DSPs, it will return a list of all protein sequences that matched both of these domains in InterProScan [113]. The types of phosphatase described in the ontology vary from the general (tyrosine phosphatase) to the specific (R2A Phosphatase). The classification process will place any phosphatase to the most specific type possible. Those proteins not fully classified are either new types not yet completely described by the ontology, or types for which there are no InterPro descriptions. To make sure no phosphatases had been missed using the ontology method, the SMART domain database [115] was scanned and sequence similarity searching was used (using BlastP programs from both NCBI and Swiss-Prot/TrEMBL [116]). Note that searches using more recent versions of InterPro may give slightly different results to those reported in this work due to updating of InterPro entries and also entries in the databases scanned by InterPro.

### Domain architecture analysis

This was performed with the information obtained from InterProScan [113]. Quality control methods were employed to distinguish true domain matches from low scoring matches. Each domain match was submitted for sequence similarity searching against the Swiss-Prot/TrEMBL database [116] to determine whether they matched other sequences with the same domain. As InterPro is a secondary domain database and searches other bioinformatics databases, the original motifs or domains descriptions and criteria were investigated for each InterPro match. For example, if the InterPro domain was based on a PRINTS fingerprint [117] entry then we investigated how many motifs of the fingerprint did actually match the query sequence. If an InterPro domain match had support from several other databases, then it was deemed to have good support and was included in this study.

### Sequence analysis of the phosphatase catalytic domains

Multiple alignments were produced with ClustalX [118] and manually edited with BioEdit [119] to obtain the most robust alignments for phylogenetic analyses. Detailed sequence analyses were performed for each kinetoplastid phosphatase subfamily. Motifs previously described as conserved in other eukaryotic catalytic domains were compared to the kinetoplastid phosphatase catalytic domains [25,26,82,91,92,120-122]. Further sequence similarity searching and motif analysis was done for the "kinatases" to analyse the features of the kinase domain and the conservation of the kinase-phosphatase domain architecture in other organisms. From BlastP searching at Swiss-Prot/TrEMBL [116], the closest annotated sequences to the kinetoplastid kinatases were found to be mammalian calcium-dependent kinase kinases (CaMKKs). A ClustalX multiple alignment was created of the kinase domains of CaMKKs from several eukaryotes (human, mouse, rat, *X. laevis*, *D. rerio*, *C. elegans*, *D. melanogaster*, *D. discoideum*, *S. cerevisiae *and *A. thaliana*) to identify conserved motifs. This alignment was manually edited and the conserved residues determined using descriptions of the 11 conserved kinase subdomains. The InterPro and SMART databases were queried to determine if kinatases were present in any other species.

Additional sequence similarity searching was performed for the DSPs containing Leucine Rich Repeats (LRRs) using BlastP [123]. The LRR regions from the six kinetoplastid sequences (LRR-DSPs and kinatases) were analysed for the closest matches in other organisms, particularly human. A BlastP search was also done for the region containing an ankyrin domain in Tc00.1047053510265.70 and the InterPro database queried for any other ankyrin phosphatase domain containing proteins.

### Phylogenetic analysis

A previously established and thorough phylogenetics approach was used [124] to produce the evolutionary trees. The different ClustalX alignments created from the phosphatase catalytic domain sequence analyses were used for the phylogenetic tree construction. All human, *S. cerevisiae *and *A. thaliana *sequences from each phosphatase subfamily were included in the alignments, unless the sequences were fragments or had deletions in important conserved regions. These sequences were included as markers and to give some functional definition to the clades in each tree determining the similarity between *T. cruzi*, *T. brucei *and *L. major *protein phosphatases with those from other eukaryotes of different complexities. BioEdit was used to manually improve the alignments and remove all gap-containing sites before the trees were created.

ClustalX neighbour-joining (NJ) trees were produced for each of the alignments and the reliability of these was tested using three separate methods: Bayesian analysis using MrBayes (version 3.1.2, [125]), maximum likelihood and maximum parsimony methods using the PHYLIP package (version 3.63) [126] and Tree-Puzzle (version 5.2, [127]. ClustalX NJ trees were used in favour of PHYLIP Neighbour-Joining as the PHYLIP NJ trees differed from results with the other three methods. This was particularly noticeable in groups of three sequences with one sequence from each parasite, as ClustalX-NJ trees, and Maximum Likelihood and Maximum Parsimony methods would group the two trypanosome sequences together with the *Leishmania *sequence less closely related, although PHYLIP's NJ methods would consistently show other orientations.

The MrBayes programme Markov Chain Monte Carlo, was used to generate an optimal tree using Bayesian methods. For Maximum Likelihood analysis, firstly gamma correction values were generated from Tree Puzzle [127]. This gives a more accurate model of amino acid substitutions as it models the rate of evolution against the frequency of sites with that rate. The value for each sequence alignment was input into the PHYLIP program, *PROML*. Global rearrangement was also used to improve the results as this option allows the programme to re-evaluate the placing of each sequence within the tree. Maximum Parsimony was performed using *SEQBOOT*, *PROTPARS *and *CONSENSE *from PHYLIP. For both ClustalX Neighbour Joining and Maximum parsimony 1000 bootstrap replicates were produced to give improved statistical values for the consensus trees. Final consensus trees were produced for every phosphatase alignment integrating results from each method. Bootstrap and Bayesian clade credibility values of 70% and above are shown in the figures.

For the MKP and lipid phosphatases phylogenetic analyses a preliminary NJ tree was produced containing all DSPs and lipid phosphatases from the three kinetoplastids, human, yeast and plant. The MKP and lipid phosphatase-containing regions were of most interest so only sequences from these groups were used for the full analysis using all four methods.

A separate analysis was performed for the kinetoplastid sequences originally identified as Low Molecular Weight PTPs (LMW-PTPs) and Cdc25. A ClustalX multiple sequence alignment included human and yeast LMW-PTP, bacterial ArsC reductases, human and mouse Cdc25 and Arc2 reductases from *S. cerevisiae *and plants, together with the putative kinetoplastids Cdc25 and LMW-PTPs. A phylogenetic tree was calculated as above.

## List of abbreviations

DSP, dual specificity phosphatase; eDSP, eukaryotic-like dual specificity phosphatase; aDSP, atypical dual specificity phosphatase; kDSP, kinetoplastid-specific dual specificity phosphatase; PTP, protein tyrosine phosphatase; kPTP, kinetoplastid-specific protein tyrosine phosphatase; PTEN, Phosphatase and tensin homolog; LMW-PTP, low molecular weight protein tyrosine phosphatase; EYA, Eyes absent phosphatase; STYX, Phosphoserine/threonine/tyrosine-binding protein; PRL, phosphatase of regenerating liver; MKP, Mitogen-activated protein kinase phosphatase; cdc25, cell division cycle phosphatase 25; MTM, Myotubularin; STP, serine/threonine phosphatase; PPP, phospho-protein phosphatase; kPPP, kinetoplastid-specific phospho-protein phosphatase; PPM, protein phosphatase, magnesium or manganese-dependent; FCP, TFIIF-stimulated CTD phosphatase; Alphs, ApaH-like phosphatases; PPEF, Protein phosphatase with EF-Hand domains; SH2, Src-homology 2; FN, Fibronectin; LRR, Leucine Rich Repeat.

## Authors' contributions

RB is responsible for data collection, phylogenetic analysis and drafting of the manuscript. JHJ contributed to the production and analysis of phylogenetic trees; HT, HM, BS contributed to several aspects of data analysis; RS is responsible for the creation of the phosphatase ontology and supervision of the computational methods; KM contributed to data analysis, biological insights and the writing of the manuscript. LT conceived the study, analysed data and wrote the manuscript. All authors read and approved the final manuscript.

## Supplementary Material

Additional file 1 **Table S1. A list of all kinetoplastid sequences described in this study**. Sequences are listed in subgroups with the systematic GeneDB ID used in this analysis. Swiss-Prot IDs are also provided. As the *T. cruzi *proteome contains sequences from two closely related species, the 'duplications' have been listed where present. 'X' means there is no duplication. InterPro domain definitions: IPR000387, Tyr-specific phosphatase; IPR000242, Protein tyrosine phosphatase; IPR003595, Protein tyrosine phosphatase – catalytic; IPR000340, Dual-specificity phosphatase; IPR000719, Protein kinase; IPR011009, Protein kinase-like; IPR001611, Leucine-rich repeat; IPR003591, Leucine-rich repeat, typical subtype; IPR002110, Ankyrin; IPR004861, Protein tyrosine phosphatase SIW14-like; IPR008973, C2 calcium/lipid-binding region CaLB; IPR014019, Phosphatase tensin type; IPR014020, C2 tensin-type; IPR010569, Myotubularin-related; IPR011011, Zinc finger FYVE/PHD-type; IPR000306, Zinc finger FYVE-type; IPR001763, Rhodanese-like; IPR006186, Serine/threonine-specific protein phosphatase and bis(5-nucleosyl)-tetraphosphatase; IPR004843, Metallophosphoesterase; IPR001440, Tetratricopeptide TPR_1; IPR011236, Protein phosphatase 5; IPR013026, Tetratricopeptide region; IPR011990, Tetratricopeptide-like helical; IPR013235, Serine/Threonine phosphatase PPP5; IPR002048, Calcium-binding EF-hand; IPR011992, EF-Hand type; IPR013105, Tetratricopeptide TPR_2; IPR015655, Protein phosphatase 2C; IPR001932, Protein phosphatase 2C-related; IPR014045, Protein phosphatase 2C N-terminal; IPR010822, Sporulation stage II, protein E C-terminal; IPR000222, PP2C manganese/magnesium aspartate binding site; IPR004274, NLI interacting factor; IPR011948, Dullard-like phosphatase. Domains highlighted in red were removed from the analysis as they had poor matches and the query sequence lacked similarity to other sequences containing the same domain: IPR000834, Peptidase M14 carboxypeptidase A; ATP-dependent helicase DEAD-box; IPR003006, Immunoglobulin MHC motif; IPR006035, Ureohydrolase; IPR001356, Homeobox; IPR000106, Protein tyrosine phosphatase low molecular weight; IPR000560, Histidine acid phosphatase; IPR013323, SIAH-type; IPR001395, Aldo/keto reductase; IPR008984, SMAD/FHA; IPR001865, Ribosomal protein S2; IPR005829, Sugar transporter superfamily; IPR001360, Glycoside hydrolase family 1; IPR002086, Aldehyde dehydrogenase. Click here for file

Additional file 2 **Table S2. PTP conserved motif analysis**. Variations from consensus sequences are highlighted in red. Click here for file

Additional file 3 **Figure S1. Synteny of PTPs in the three kinetoplastids**. Comparison of the corresponding syntenic regions from the *L. major, T. cruzi *and *T. brucei *genomes around *Lm*PTP1. Analyses were conducted via TBlastX using the Artemis comparison tool [132] with an E value of and default Gap settings. Outputs were manually annotated using GeneDB annotations. Click here for file

Additional file 4 **Table S3. Kinetoplastid-specific motifs in TriTryp classical Tyr-specific PTPs**. Sequence motifs conserved in kinetoplastid PTPs but not in other eukaryotes. PcT1 and PcT2 are located at the N-terminal region before the phosphatase catalytic domain. T1–T4 are located in the phosphatase catalytic domain. Click here for file

Additional file 5 **Table S4. Atypical DSP motif analysis**. Key functional residues are in bold in the column headings. All substitutions from the classic DSP motifs are highlighted in red. Click here for file

Additional file 6 **Figure S2 Phylogenetic tree of LMW-PTPs, mammalian Cdc25 with arsenate reductases type ArsC and ACR2**. Sequences were inferred using the Neighbour-Joining method of PHYLIP. LMW-PTPs from *Homo sapiens *(HUMAN_P24666) and *Saccharomyces cerevisiae *(YEAST_P40347) are shown. ArsC reductases are included from *Bacillus subtilis *(BACSU_P45947) and *Staphylococcus aureus *(STAAU_P0A006). Kinetoplastid sequences found grouping with these ArsC reductases include *T. brucei *(Tb09.160.2100), *T. cruzi *(Tc00.1047053504797.120), and *L. major *(LMJ_0020). CDC25s from *H. sapiens *(HUMAN_P30307) and *Rattus norvegicus *(RAT_P48966) are shown. A second group of kinetoplastid sequences including *Leishamania infantum *(LEIIN_A4I895), *L. major *(LmjF32.2740), *Leishmania braziliensis *(LbrM32_V2.2980), and *T. cruzi *(Tc00.1047053508707.20) were found to group with the ACR2 reductases from *S. cerevisiae *(YEAST_Q06597), and the plants *Arabidopsis thaliana *(ARATH_Q8GY31), and *Oryza sativa *(ORYSJ_Q9AV34). The tree is unrooted and the scale indicating amino acid replacements per site is shown. Click here for file

Additional file 7 **Figure S3. Synteny of Cdc25-like Acr2 between *L. major *and *T. brucei***. Comparison of the corresponding syntenic regions from the *L. major *and *T. brucei *genomes around the ACR2 phosphatase (LmjF32.2740). Analyses were conducted via TBlastX using the Artemis comparison tool [132] with an E value of 1.00 and default Gap settings. Outputs were manually annotated using GeneDB annotations. Click here for file

Additional file 8 **Table S5. Motif analysis for the PPP family of Ser/Thr phosphatases**. Residues in red are substitutions from the conserved pattern: G**D**X**H**G – G**D**XVDRG – G**N**HE[82] (residues in bold coordinate metal ions at the catalytic site and the underlined His is the proton donor in catalysis). Click here for file
