# Supplementary figures and images for: The TriTryp Phosphatome: analysis of the protein phosphatase catalytic domains
Source: BMC Genomics. 2007 Nov 26;8:434. doi: 10.1186/1471-2164-8-434 (PMC2175518; doi:10.1186/1471-2164-8-434)

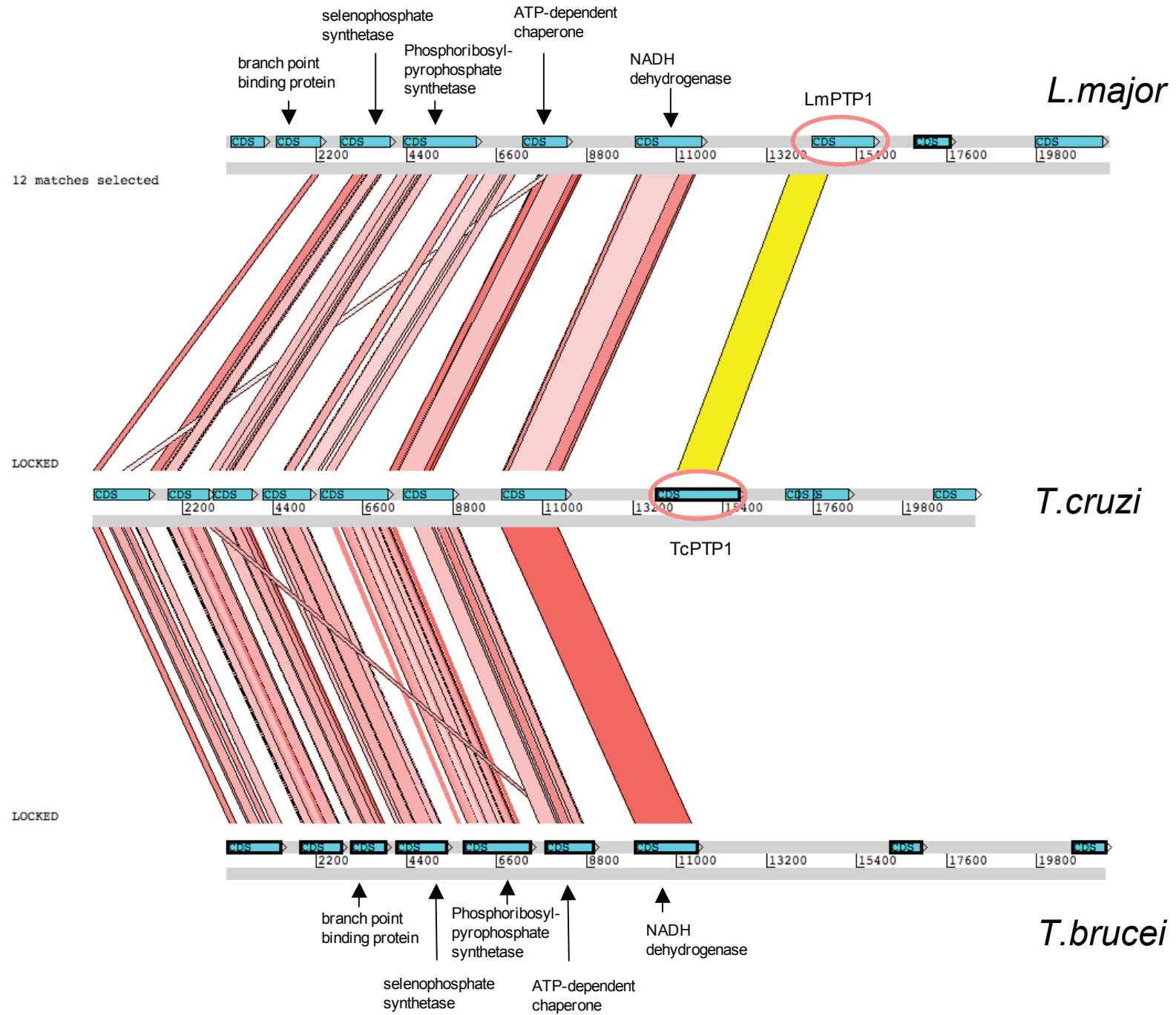

Supplement: Additional file 3 — Figure S1. Synteny of PTPs in the three kinetoplastids. Comparison of the corresponding syntenic regions from the L. major, T. cruzi and T. brucei genomes around LmPTP1. Analyses were conducted via TBlastX using the Artemis comparison tool [132] with an E value of and default Gap settings. Outputs were manually annotated using GeneDB annotations. [file 1471-2164-8-434-S3.pdf]

ACR2 Reductase

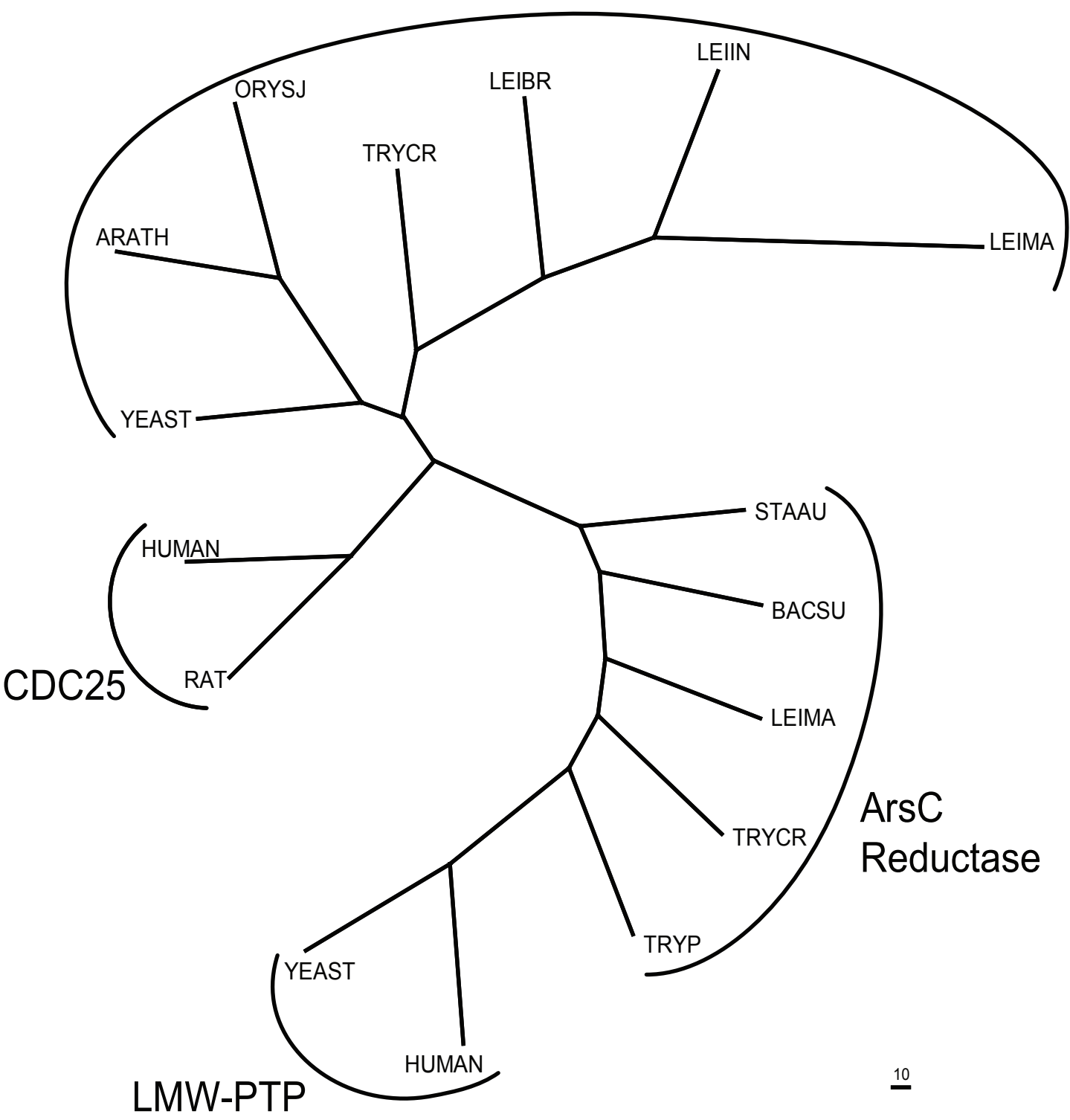

Supplement: Additional file 6 — Figure S2 Phylogenetic tree of LMW-PTPs, mammalian Cdc25 with arsenate reductases type ArsC and ACR2. Sequences were inferred using the Neighbour-Joining method of PHYLIP. LMW-PTPs from Homo sapiens (HUMAN_P24666) and Saccharomyces cerevisiae (YEAST_P40347) are shown. ArsC reductases are included from Bacillus subtilis (BACSU_P45947) and Staphylococcus aureus (STAAU_P0A006). Kinetoplastid sequences found grouping with these ArsC reductases include T. brucei (Tb09.160.2100), T. cruzi (Tc00.1047053504797.120), and L. major (LMJ_0020). CDC25s from H. sapiens (HUMAN_P30307) and Rattus norvegicus (RAT_P48966) are shown. A second group of kinetoplastid sequences including Leishamania infantum (LEIIN_A4I895), L. major (LmjF32.2740), Leishmania braziliensis (LbrM32_V2.2980), and T. cruzi (Tc00.1047053508707.20) were found to group with the ACR2 reductases from S. cerevisiae (YEAST_Q06597), and the plants Arabidopsis thaliana (ARATH_Q8GY31), and Oryza sativa (ORYSJ_Q9AV34). The tree is unrooted and the scale indicating amino acid replacements per site is shown. [file 1471-2164-8-434-S6.pdf]
