# Supplementary material for: The TriTryp Phosphatome: analysis of the protein phosphatase catalytic domains
Source: BMC Genomics. 2007 Nov 26;8:434. doi: 10.1186/1471-2164-8-434 (PMC2175518; doi:10.1186/1471-2164-8-434)
Supplement: Additional file 4 — Table S3. Kinetoplastid-specific motifs in TriTryp classical Tyr-specific PTPs. Sequence motifs conserved in kinetoplastid PTPs but not in other eukaryotes. PcT1 and PcT2 are located at the N-terminal region before the phosphatase catalytic domain. T1–T4 are located in the phosphatase catalytic domain. [file 1471-2164-8-434-S4.doc]

**Table S3. Kinetoplastid-specific motifs in TriTryps classical PTPs.**

| **Systematic ID** | **Name** | **N-ter**  **Extension**  **(aa)** | **C-ter**  **Extension**  **(AA)** | **Kinetoplastid-Specific Motifs** | | | | | |
| --- | --- | --- | --- | --- | --- | --- | --- | --- | --- |
|  |  |  |  | **PcT1** | **PcT2** | **T1** | **T2** | **T3** | **T4** |
| Tb10.70.0070 | TbPTP1 | **-** | **-** | QREFVQL | QENPRNINFTT | LANEATL | YEV | LIGAYAA | RLGVDI |
| Tc00.1047053510187.234 | TcPTP2 | **-** | **-** | QREFSLL | QEDPRSISFAT | LANEATL | YEV | LIGAYAA | RLGVDI |
| LmjF36.2180 | LmjPTP2 | 60 | **-** | EEEFAAI | TMNPRLYNFTT | LANEETI | **-** | LIGAYTA | HMGVDA |
| Tc00.1047053506839.60 | TcPTP1 | **-** | 214 | GFDEEMD | RSNVDEF | RANEGT | **-** | FIALHVA | EMDRMI |
| LmjF36.5370 | LmjPTP1 | **-** | - | GFDRELA | RSNLDEY | KPNEGT | **-** | FIGLHIA | EMERMI |
