# Supplementary material for: The TriTryp Phosphatome: analysis of the protein phosphatase catalytic domains
Source: BMC Genomics. 2007 Nov 26;8:434. doi: 10.1186/1471-2164-8-434 (PMC2175518; doi:10.1186/1471-2164-8-434)
Supplement: Additional file 5 — Table S4. Atypical DSP motif analysis. Key functional residues are in bold in the column headings. All substitutions from the classic DSP motifs are highlighted in red. [file 1471-2164-8-434-S5.doc]

**Table S4. Atypical DSPs conserved motif analysis.**

|  | **Sequence** | **D-loop**  (I/V/L)X_3_**D**X_5_(I/L) | **P-loop**  VH**C**X_2_G(VI)S**R**S | **AYLM** | **R-motif**  **R**X_4_**PN**X**_2_**FX**_2_QL** |
| --- | --- | --- | --- | --- | --- |
| 1 | Tc00.1047053504033.170 | LCPE**E**YAESN**V** | **I**HCNKG**KH**R**T** | **X** | **G**DKAR**MG**DQ**Q**YV**E**L |
| 1 | Tb09.v1.0350 | LCPEDYAESN**V** | **I**HCNKG**KH**R**T** | **X** | **G**DKVR**MG**DM**Q**YV**E**L |
| 1 | LmjF04.0560 | LCPEDYAESNL | **I**HCNKG**KH**R**T** | **X** | **S**DKAR**VG**DQ**Q**YV**E**L |
| 2 | Tc00.1047053507093.330 | LLTE**S**HDEAF**V** | **LT**CSVG**RY**R**T** | **X** | **Q**DKGR**AE**NE**E**FI**E**L |
| 2 | Tb11.03.0500 | LLTD**S**HDEAF**V** | **LT**CSMG**RY**R**T** | **X** | **Q**DKSR**AD**NE**E**FI**E**L |
| 2 | LmjF25.0570 | LLTD**I**HDEAF**V** | **IT**CSKG**RY**RS | **X** | **G**NKSR**AD**NE**E**FI**E**L |
| 3 | LmjF33.2840 | **X** | **I**HCTAG**KD**R**T** | **D**YL**L** | **M**TAIW**TV**FD**E**YL**DA** |
| 3 | LmjF22.0250 | **X** | **F**HCTAG**KD**R**T** | **X** | **V**DLLF**RA**QAFFL**E**L |
| 4 | Tc00.1047053511725.240 | **X** | **FT**CPTG**DIQT** | **X** | **X** |
| 4 | Tb11.01.5870 | **X** | **FT**CPTG**ELQT** | **X** | **X** |
| 4 | LmjF32.1010 | **X** | **FA**CPLG**ELQT** | **X** | **X** |
| 5 | Tb09.211.0210 | **S**LTP**E**EPTYDL | **I**HCLDG**RHTT** | **X** | **W**NVQD**EV**AF**I**AD**YS** |
| 5 | LmjF04.0840 | **Y**LIP**E**PPTYDL | **I**HCLDG**RHVT** | **X** | REVQD**EA**AF**I**AD**YT** |
| 6 | Tc00.1047053506977.10 | AVMFDSADENI | VH**S**HFG**L**SRS | AY**FI** | **H**KDMC**IK**PHFQRQL |
| 6 | Tb10.70.6300 | AVMFDSADEN**V** | VH**S**QLG**M**SRS | AYL**I** | **H**KDMS**IK**PHFLRQL |
| 6 | LmjF21.0700 | **T**ILFDAGDDNI | VH**S**VYG**N**SRS | AY**MI** | **H**PDSD**IK**PHFQRQL |
