# Supplementary material for: The TriTryp Phosphatome: analysis of the protein phosphatase catalytic domains
Source: BMC Genomics. 2007 Nov 26;8:434. doi: 10.1186/1471-2164-8-434 (PMC2175518; doi:10.1186/1471-2164-8-434)
Supplement: Additional file 7 — Figure S3. Synteny of Cdc25-like Acr2 between L. major and T. brucei. Comparison of the corresponding syntenic regions from the L. major and T. brucei genomes around the ACR2 phosphatase (LmjF32.2740). Analyses were conducted via TBlastX using the Artemis comparison tool [132] with an E value of 1.00 and default Gap settings. Outputs were manually annotated using GeneDB annotations. [file 1471-2164-8-434-S7.pdf]

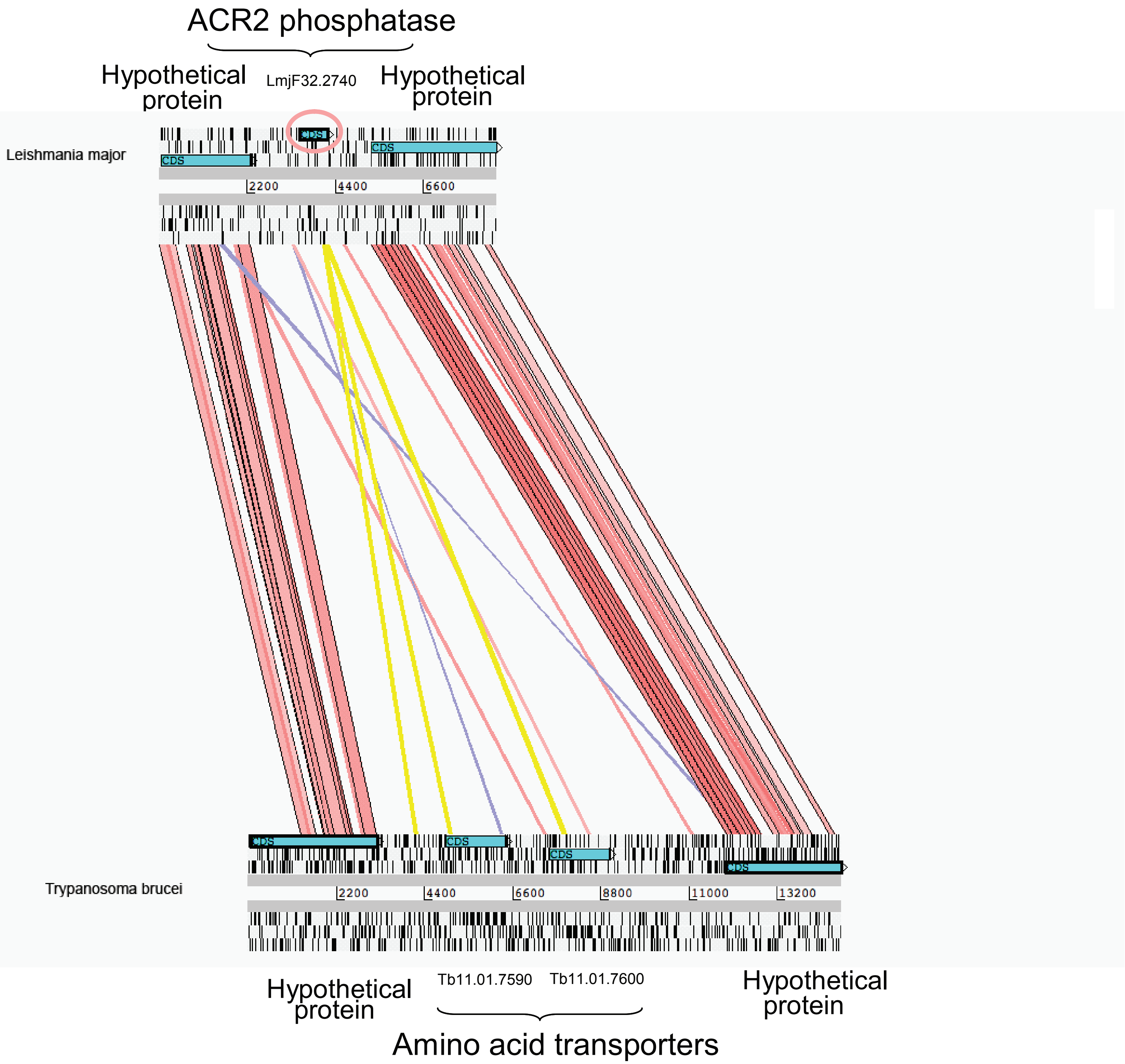
ACR2 phosphatase
Hypothetical protein
LmjF32.2740
Hypothetical protein
Leishmania major
CDS
CDS
CDS
2200
4400
6600
CDS
CDS
CDS
CDS
Trypanosoma brucei
2200
4400
6600
8800
11000
13200
Hypothetical protein
Tb11.01.7590
Tb11.01.7600
Hypothetical protein
Amino acid transporters
