# Supplementary material for: The TriTryp Phosphatome: analysis of the protein phosphatase catalytic domains
Source: BMC Genomics. 2007 Nov 26;8:434. doi: 10.1186/1471-2164-8-434 (PMC2175518; doi:10.1186/1471-2164-8-434)
Supplement: Additional file 8 — Table S5. Motif analysis for the PPP family of Ser/Thr phosphatases. Residues in red are substitutions from the conserved pattern: GDXHG – GDXVDRG – GNHE[82] (residues in bold coordinate metal ions at the catalytic site and the underlined His is the proton donor in catalysis). [file 1471-2164-8-434-S8.doc]

**Table S5. Motif analysis for the PPP family of Ser/Thr phosphatases**.

|  | **Accession No.** | G**D**X**H**G | G**D**XV**DR**G | G**NH**E | **Subfamily** |
| --- | --- | --- | --- | --- | --- |
| **Group 1 – Alphs** | Tc00.1047053506489.50 | GDIHG | GDMV**NK**G | GNH**D** | kPPP |
|  | Tc00.1047053504411.30 | GDIHG | GDMV**NK**G | GNH**D** | kPPP |
|  | Tc00.1047053509211.30 | GDVHG | GDLV**NK**G | GNH**D** | kPPP |
|  | Tb927.4.4330 | GDIHG | GDLV**NK**G | GNH**D** | kPPP |
|  | Tb927.6.640 | GDVHG | GDLV**NK**G | GNH**D** | kPPP |
|  | Tb927.8.8040 | GDIHG | GDLV**NK**G | GNH**D** | kPPP |
|  | LmjF17.0580 | **PGPL**G | GDLV**NK**G | GNH**D** | kPPP |
|  | LmjF22.1600 | GDVHG | GDYV**NK**G | GNH**D** | kPPP |
| **Group 2 – Other catalytic mutations** | Tc00.1047053510187.500 | GDI**R**G | G**N**Y**T**DR**S** | GNHE | PP2B |
|  | Tc00.1047053506833.30 | GDLHG | GDI**L**D**V**G | GNHE | kPPP |
|  | Tb10.70.0350 | GDV**R**G | G**N**Y**I**D**SC** | GNHE | PP2B |
|  | Tb927.6.1230 | G**P**C**R**G | G**N**YVD**G**G | G**R**HE | kPPP |
|  | LmjF12.0050 | G**P**I**R**G | G**N**Y**I**D**GA** | G**K**HE | kPPP |
|  | LmjF24.0270 | GDL**L**G | G**N**YVD**V**G | G**P**HE | kPPP |
|  | LmjF29.0440 (LMJ_0679) | GDLHG | G**N**VV**GYG** | GN**Q**E | kPPP |
|  | LmjF30.3280 | GDLHG | GDI**L**D**V**G | GNHE | kPPP |
|  | LmjF36.1980 | GDV**Q**G | G**N**Y**IGN**G | G**S**NE | PP2B |
